# Cryopreservation of Medicinal Plant Seeds: Strategies for Genetic Diversity Conservation and Sustainability

**DOI:** 10.3390/plants13182577

**Published:** 2024-09-13

**Authors:** Lin Zeng, Zheng Sun, Li Fu, Yakun Gu, Rongtao Li, Mingjun He, Jianhe Wei

**Affiliations:** 1 Hainan Provincial Key Laboratory of Resources Conservation and Development of Southern Medicine, Hainan Branch of the Institute of Medicinal Plant Development, Chinese Academy of Medical Sciences and Peking Union Medical College, Haikou 570311, China; zzz1136134838@163.com (Z.S.); 18789701748@163.com (L.F.); guyakun0920@126.com (Y.G.); lirt99@126.com (R.L.); hmj2008@163.com (M.H.); 2 Key Laboratory of Bioactive Substances and Resources Utilization of Chinese Herbal Medicine, Institute of Medicinal Plant Development, Chinese Academy of Medical Sciences and Peking Union Medical College, Beijing 100193, China

**Keywords:** medicinal plant, cryopreservation, biodiversity conservation, cryobank, recalcitrant seeds

## Abstract

The depletion of medicinal plant resources leads to the irreversible loss of their genetic diversity. The preservation of medicinal plant germplasm using cryobanks is crucial for maintaining the sustainability of these resources. This study examined the efficacy of cryopreservation on 164 medicinal plant seeds, identified general principles for preserving medicinal plant seeds at ultra-low temperatures, and established a cryobank for dry-sensitive medicinal plant seeds. Over 90% of orthodox seeds were unaffected by freezing, with optimal conditions being a 5–10% moisture content and direct freezing. Intermediate seeds were best frozen with a 7–15% moisture content, and those with a lower initial moisture content were best suited to direct freezing. While recalcitrant seeds’ freezing was most influenced by moisture content, there was no specific range. Direct freezing is appropriate for recalcitrant seeds possessing a hard seed coat and a firm seed kernel, whereas seeds with a brittle or soft seed coat are better suited for vitrification or stepwise freezing methods. There was no significant correlation between alterations in physiological and biochemical indicators and microscopic structures of seeds before and following liquid nitrogen freezing, as well as their storage characteristics. The findings of this research offer evidence in favor of the extended conservation of plant seeds and the extensive utilization of ultra-low temperature technology and provides an example of protecting the genetic diversity of plant resources.

## 1. Introduction

According to data from the World Health Organization, an estimated 75% of the global population utilizes plant-based remedies for health preservation, with approximately 21,000 plant species recognized for their medicinal properties [1]. Despite advancements in synthetic compounds, botanical sources continue to serve as the predominant reservoir of medicinal agents on a global scale [2]. The decline in the diversity of medicinal plant resources can be attributed to the impact of human activities and climate change. This has resulted in numerous species facing the threat of extinction, thereby jeopardizing crucial natural resources essential for safeguarding human health [3]. Additionally, this decline has hampered efforts to conserve plant genetic diversity through in situ conservation measures [4]. The preservation and sustainable management of genetic resources of medicinal plants should be regarded as a paramount concern in order to fulfill the needs of forthcoming generations [2]. Hence, ex situ conservation, which is considered a primary approach for safeguarding plant genetic resources, has been extensively utilized, predominantly through the establishment of seed gene banks. Seeds possess substantial genetic variability and serve as the principal medium for plant propagation, making them an invaluable resource for the preservation of species diversity [5]. Currently, the gene bank method stands as the most cost-effective and secure approach for the strategic conservation of biological resources. Plant germplasm resource banks safeguard plant germplasm resources, and low-temperature germplasm resource banks have been demonstrated to be the optimal preservation method. Currently, there exist over 1700 germplasm banks of diverse plant taxa worldwide. These repositories have amassed a collection of over 7.4 million plant germplasm resources, with approximately 90% of these resources being conserved as seed bodies within low-temperature germplasm banks.

A diverse set of medicinal plant seeds with unique biological characteristics exists, which necessitates a variety of storage conditions. Based on the storage attributes of seeds, medicinal plant seeds can be categorized into three groups: orthodox seeds, intermediate seeds, and recalcitrant seeds. Presently, the majority of international seed gene banks predominantly conserve desiccated seeds [6], a method that is generally applicable to most orthodox seeds. Nonetheless, certain orthodox seeds experience a notable decline in vitality following a period of low temperature and low humidity lasting from 3 to 5 years. Conversely, seeds that are sensitive to desiccation, as well as intermediate and recalcitrant seeds, are unable to endure water loss and thus cannot be accommodated within traditional seed gene banks [7,8]. A cryobank for medicinal plants (National Southern Medicinal Gene Bank) has been established in Hainan Province, China, with the purpose of preserving dehydration-sensitive medicinal plant seeds in cryopreservation with liquid nitrogen. Utilizing cryopreservation, the cryobank has successfully preserved 11,055 medicinal plant seeds.

Cryopreservation has emerged as the optimal approach for the extended preservation of desiccation-sensitive seeds [9,10], entailing the placement of seeds in either liquid nitrogen (−196 °C) or its vapor phase (−140 °C). Under such extreme temperatures, the metabolic activity and cellular division of plant seeds essentially halt, rendering them in a state of “pseudo-death”, thereby facilitating long-term storage [11,12]. Simultaneously, cryopreservation serves as a preventive measure against subsequent cultivation and the accumulation of mutations in natural environments. Thus, it is the most advantageous and secure technique for conserving plant seeds and averting their deterioration, thereby guaranteeing the secure preservation of genetic resources pertaining to valuable plant species [13]. The application of this method has proven effective in safeguarding numerous species within the realms of agriculture [14,15,16,17], horticulture [18,19,20,21], and forestry [22,23,24,25] as well as certain medicinal plant germplasms [26]. The authors have successfully implemented ultra-low temperature preservation techniques on the seeds of medicinal plants, including *Dalbergia odorifera* T. Chen [27], *Alpinia oxyphylla* Miq. [28], *Myristica fragrans* Houtt. [29], and *Prunella vulgaris* L. [30], during the initial phase of their research. However, given the unique characteristics of recalcitrant and intermediate seeds that lack resistance to desiccation and low temperatures, it has become imperative to individually employ cryopreservation techniques, a process that can be time-consuming. Additionally, certain recalcitrant seeds may lose their viability before the completion of the cryopreservation treatment. Moreover, in the case of bulk seed collection, it is essential to swiftly and efficiently carry out mass preservation to prevent seed wastage.

This study aimed to determine the effectiveness of cryopreservation for medicinal plant seeds by conducting liquid nitrogen ultra-low temperature preservation experiments on the seeds of 164 species of medicinal plants from 65 families in southern China. The freezing methods and viability recovery capabilities of these seeds were evaluated through the detection of seed physiological and biochemical indicators and the observation of seed microstructure.

## 2. Results

### 2.1. Seed Storage Characteristics Results

**Figure 1:**
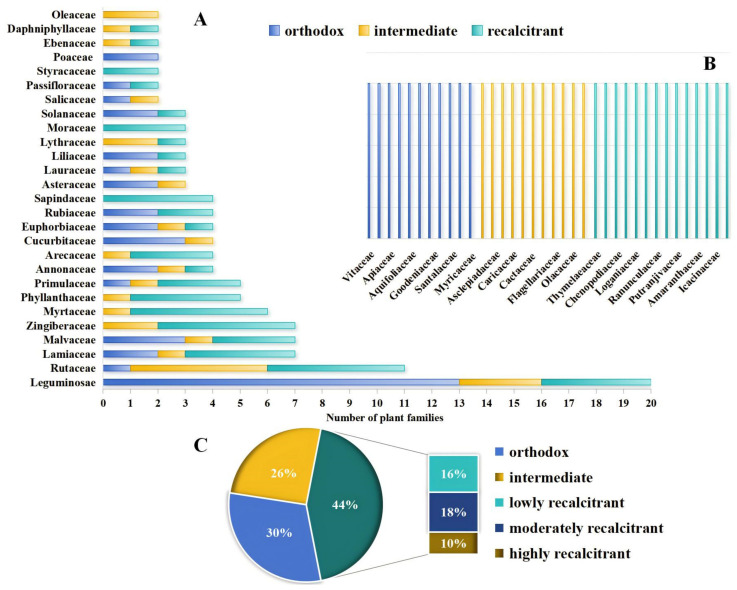
Data analysis chart of seed storage characteristics results. Note: (**A**,**B**) show the distribution of plant families for testing seed storage properties. (**A**) shows at least two plants in a family, and (**B**) shows a single plant. (**C**) shows the proportion of storage characteristics of various types of seeds.

Based on the evaluation method and steps for seed storage characteristics, a total of 164 medicinal plant seeds were categorized into three groups: 50 orthodox seeds, 42 intermediate seeds, and 72 recalcitrant seeds (Figure 1 or Table A1). The statistical results presented in Figure 1A,B indicate that the orthodox seeds were predominantly from families such as *Leguminosae*, *Poaceae*, *Cucurbitaceae*, and *Malvaceae* and were characterized by their small seed size. Intermediate seeds were primarily associated with plants from the *Rutaceae* and *Liliaceae* families. Recalcitrant seeds, in contrast, were largely collected from tropical and subtropical regions, such as Hainan, Guangdong, and Yunnan, and were primarily represented by plants from the *Arecaceae, Zingiberaceae*, *Myrtaceae,* and *Sapindaceae* families that are known for their large seed size. The seed storage characteristics within a given genus of plants may vary. For example, *H. mutabilis*, belonging to the *Hibiscus* genus of the *Malvaceae* family, displays recalcitrant seed behavior, whereas *H. sabdariffa* exhibits normal seed characteristics. Similarly, *V. trifolia*, a member of the *Vitex* genus within the *Lamiaceae* family, has orthodox seeds, while *V. quinata* has recalcitrant seed properties.

The seeds of *A. heterophyllus*, *A. catechu*, and other similar species exhibited a high level of recalcitrance when dehydrated to a moisture content of 30%. This dehydration significantly diminished their viability and rendered them poorly tolerant to the absence of water. Consequently, these seeds were classified as highly recalcitrant. Conversely, the seeds of *P. pseudoginseng*, *E. lepta*, and other comparable species maintained a considerable level of viability, even when dehydrated to a moisture content ranging from 15 to 30%. The seeds exhibited a moderate tolerance to dehydration, and these seeds could thus be categorized as moderately recalcitrant. Seeds of *A. sinensis*, *A. katsumadai,* and other species exhibited a minor decline in viability as the moisture content decreased, if this remained above 12%. Additionally, these seeds could be stored within a temperature range of 4–10 °C for a specific duration and thus possessed characteristics of low recalcitrance. The statistical results presented in Figure 1C reveal the classification of the experimental seeds in this study as follows: 16 seeds exhibit high recalcitrance, 29 seeds display moderate recalcitrance, and 27 seeds demonstrate low recalcitrance.

### 2.2. Cryopreservation of Seeds

**Figure 2:**
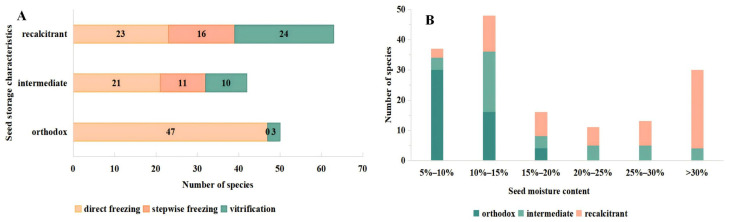
Analysis chart of seed cryopreservation results. Note: (**A**) shows the distribution of suitable freezing methods for various types of seeds that have been successfully frozen. (**B**) shows the distribution of seed moisture content ranges for successful freezing of various types of seeds.

Seeds from 164 species of medicinal plants were subjected to ultra-low temperature freezing, resulting in the successful freezing of 155 species (Figure 2 or Table A1). Both orthodox and intermediate seeds were effectively frozen. However, for nine recalcitrant seeds, *H. coronarium*, *S. cumini*, *S. aromaticum*, *S. tonkinensis*, *P. nigrum*, *B. balansae*, *M. falcatum*, *C. liberica*, and *S. nervosum*, their viability was below 30% or even non-existent following freezing. Based on the analytical data presented in Figure 2A, it was evident that out of the 155 seeds that were successfully frozen, 91 seeds were most effectively preserved through direct freezing, comprising 47 orthodox seeds, 21 intermediate seeds, and 23 recalcitrant seeds. Additionally, 27 seeds (11 intermediate seeds and 16 recalcitrant seeds) were determined to have achieved optimal preservation through stepwise freezing. Furthermore, 37 seeds attained the highest level of preservation through vitrification, comprising three orthodox seeds, 10 intermediate seeds, and 24 recalcitrant seeds.

Recalcitrant seeds with hard seed coats and firm kernels such as *A. catechu*, *M. citrifolia*, *S. lanceolata*, *S. lychnophora*, *S. maritima* and *V. negundo* exhibit enhanced viability when subjected to direct freezing in liquid nitrogen compared to other groups. Conversely, for recalcitrant seeds possessing crisp or soft seed coats, such as *O. pinnata*, *S.dulcificum*, *A. obtusa, A. heterophyllus*, *D. calycinum* and *L. inermis*, vitrification or stepwise freezing methods have been demonstrated to be the most suitable freezing techniques.

Observing Figure 2B, we can determine that the viability of most orthodox seeds was not significantly different before and after freezing, provided that their moisture content remained below 15%. However, when the moisture content exceeded 15%, the viability of the seeds decreased after freezing compared to their no-freezing state. Consequently, the recommended moisture content range for cryopreservation was generally between 5 and 15%. Furthermore, the orthodox seeds subjected to freezing using the three different methods displayed enhanced vitality. Notably, the seeds that underwent direct freezing exhibited higher vitality than those subjected to stepwise freezing or vitrification. The viability of intermediate seeds was lower in response to dehydration and low temperature in comparison to orthodox seeds. Additionally, the majority of seeds exhibited reduced viability following cryopreservation. However, the seeds of *C. maxima*, *D. kaki*, *P. granatum*, *A. esculentus*, *P. perfoliatum*, and *T. scabra* demonstrated higher viability after cryopreservation, specifically within the moisture content range of 7–15%.

Seeds exhibiting recalcitrant behavior demonstrated heightened susceptibility to cryopreservation, with their viability significantly impacted by moisture levels. Notably, 15 seeds with low recalcitrance, including *A. sinensis* and *A. katsumadai*, exhibited robust vitality following cryopreservation within the moisture content range of 12–20%. Additionally, the seeds of *A. muricarpum*, *V. negundo,* and *C. argentea* displayed increased vitality after cryopreservation compared to their no-freezing state. Seeds with moderate and high levels of recalcitrance exhibited heightened susceptibility to freezing when frozen using liquid nitrogen. In general, seeds with a moisture content below 30% demonstrated diminished viability following freezing, except for those falling within the moisture content range of 35 to 50%. Nevertheless, the seeds of *S. lanceolata*, *S. myrsinifolium*, *D. calycinum*, *E. japonica*, and other species within each moisture content range displayed vitality levels below 30%.

### 2.3. Detection of Physiological and Biochemical Indicators of Seeds

**Figure 3:**
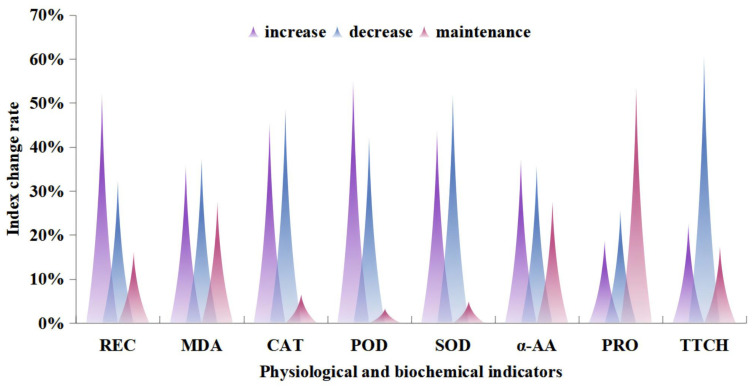
Analysis chart of changes in physiological and biochemical indicators of seeds before and after cryopreservation. Note: REC: relative conductivity; MDA: malondialdehyde; CAT: catalase; POD: peroxidase; SOD: superoxide dismutase; α-AA: α-amylase; PRO: soluble protein; TTCH: dehydrogenase.

After subjecting 62 medicinal plant seeds to ultra-low temperature freezing with liquid nitrogen, a comprehensive analysis was conducted on various parameters, including α-amylase activity, dehydrogenase activity, MDA content, SOD activity, POD activity, CAT activity, protein content, and conductivity (Figure 3 or Table A2). The results revealed changes in these parameters following the cryopreservation process. Among the seeds analyzed, 64.52% exhibited an increase or maintenance in α-amylase activity, while 60.34% displayed a decrease in dehydrogenase activity. Additionally, 48.39% of the seeds exhibited a decrease in CAT activity compared to their pre-freezing state, whereas 43.55% exhibited an increase in SOD activity. Furthermore, 53.24% of the seeds showed no significant change in protein content compared to the control group. The changes in these index values were not significantly related to seed storage characteristics.

Of the seeds subjected to cryopreservation in liquid nitrogen, the structural integrity of the seed cell membranes was compromised in 51.79%. The freezing process involving liquid nitrogen potentially resulted in the disruption of the enzyme system in approximately 35.48% of the seeds. Additionally, this process caused a significant buildup of MDA content within the seed cells, particularly in recalcitrant seeds, as these comprised 50%. The application of liquid nitrogen freezing resulted in a notable improvement in the capacity of 54.84% of the seeds to eliminate free radicals. Moreover, the POD activity exhibited a significant increase following the freezing process.

### 2.4. Observation of Seed Microstructure

**Figure 4:**
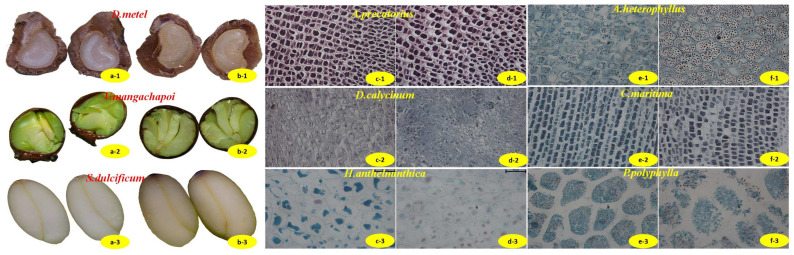
Examples of microstructural observation results of seeds before and after freezing. Note: (**a**) cross-section view of control seeds, (**b**) cross-section view of frozen seeds ((**b-1**): lackluster appearance, (**b-3**): browning and (**b-2**): dehydrated), (**c**) control seed embryo cells, (**d**) frozen seed embryo cells ((**d-1**): plasmolysis, (**d-2**): irregular arrangement and (**d-3**): loose organelles), (**e**) control seed endosperm cells, (**f**) frozen seed endosperm cells ((**f-2**): changes in content, (**f-1**): increase or (**f-3**): decrease in the number of starch granules).

**Figure A1:**
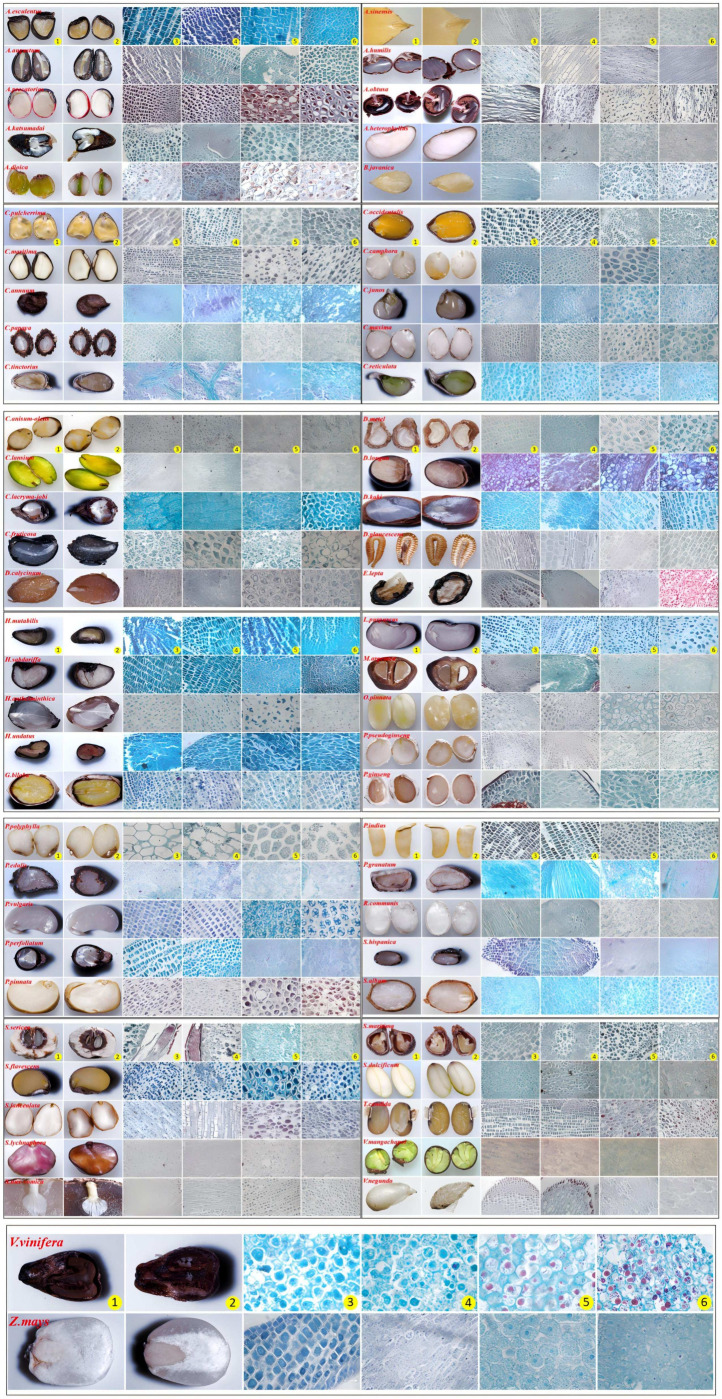
Microstructural observation results of seeds before and after freezing. Note: 1 Cross-section view of control seeds, 2 Cross-section view of frozen seeds, 3 Control seed embryo cells, 4 Frozen seed embryo cells, 5 Control seed endosperm cells, 6 Frozen seed endosperm cells.

A total of 62 seed types were subjected to cross-section and paraffin sectioning for microscopic examination both before and after undergoing ultra-low temperature freezing (Figure 4 or Figure A1). This sample included 20 seeds classified as orthodox, 17 seeds classified as intermediate, and 25 seeds classified as recalcitrant. The results showed that the majority of seeds that exhibited no apparent alterations in their cross-sectional surfaces following cryopreservation were those with low water content. Specifically, this category encompassed nine orthodox species, three intermediate species, and six low-refractory species, a proportion of 29.03%. The transverse surface of the remaining frozen seeds exhibited a lackluster (Figure 4(b-1)) appearance characterized by a brown (Figure 4(b-3)), dehydrated, and cracked texture (Figure 4(b-2)). Nevertheless, the freezing process utilizing liquid nitrogen did not have any discernible impact on the appearance of the majority of seeds.

Seeds showed plasmolysis before and after freezing with liquid nitrogen. After freezing, plasmolysis was more pronounced in seven types of seeds, including *A. precatorius* (Figure 4(d-1)). Approximately 27.42% of seed cells displayed irregular arrangement (Figure 4(d-2)) or slight deformation post-freezing, in contrast to their pre-freezing state. Internal organelles were loosely distributed (Figure 4(d-3)) in 18 types of seed embryo cells, and 64.52% of seeds showed changes in inclusions (Figure 4(f1–f3)) after freezing. However, the changes in cell arrangement and content did not demonstrate a significant correlation with seed storage characteristics.

### 2.5. Seed Germination Results

A germination experiment was conducted on a total of 50 seed varieties that had been subjected to freezing in liquid nitrogen (Table 1). The results revealed that out of the 13 orthodox seed types, all germinated, and the germination rates were above 50%, except for *S. sericea*, *M. argentea*, and *C. lacryma-jobi*. Additionally, intermediate seeds were capable of germination. Notably, *H. undatus*, *A. esculentus*, and *C. papaya* seeds demonstrated higher germination rates, while the remaining 10 seed varieties exhibited rates < 30%. For the recalcitrant seeds, a total of eight species, namely *C. pulcherrima*, *H. campechianum*, *S. lanceolata*, *C. annuum*, *L. inermis*, *A. sinensis*, *S. nux-vomica*, and *C. argentea*, successfully germinated. However, 15 species, including *S. lychnophora*, *C. lansium*, and *A. catechu*, exhibited either negligible germination or an exceptionally low germination rate.

Among the seeds examined, those classified as low recalcitrant such as *A. sinensis* exhibited germination rates exceeding 40%. Conversely, the germination rates of moderately recalcitrant seeds, including *S. dulcificum*, were relatively low. Notably, except for *A. catechu*, highly recalcitrant seeds displayed minimal or no germination. Furthermore, a majority of seeds with low or no germination rates exhibited browning and mold growth. While seed vitality is theoretically positively associated with germination rates, the process of seed germination is intricate and multifaceted.

## 3. Discussion

### 3.1. Appropriate Moisture Content and Freezing Method Contribute to the Success of Seed Cryopreservation

The freezing treatment plays a pivotal role in the ultra-low temperature preservation process, where a decrease in the preservation temperature increases the likelihood of ice crystal formation in the cells. Given the variation in water content, physiological status, and other characteristics of plant materials, diverse freezing methods are necessary. Plant materials with a low water content, such as pollen [31], orthodox seeds [13,32], some intermediate seeds [33], and a few recalcitrant seeds [30], can be subjected to direct immersion in liquid nitrogen without the need for intricate dehydration and freezing protection agents. This rapid cooling process significantly minimizes the time required for ice nucleation, thereby preventing intracellular crystallization and reducing cellular freezing damage. Consequently, cryopreservation is effectively achieved [34].

However, it is necessary for plant stem tips [35], callus tissue, somatic embryos [36], and high-moisture freezing materials such as recalcitrant seeds to undergo proper dehydration or be subjected to appropriate cryoprotectant treatment prior to their immersion in liquid nitrogen. This process allows for the freezing material to be vitrified and minimizes the formation of ice crystals [37]. Vitrification freezing, a highly prevalent cryopreservation technique, is applicable for freezing materials characterized by high moisture content, including seed embryos and plant stem tips. Moreover, this method is also well-suited for seeds with elevated moisture levels. Additionally, stepwise freezing, facilitated by the incorporation of cryoprotectants to achieve vitrification, is deemed suitable for seeds with high moisture content. Among the 155 species that were successfully subjected to cryopreservation in this study, seeds possessing comparatively lower moisture content were better suited for direct freezing, whereas intermediate or recalcitrant seeds with higher moisture content were more appropriate for vitrification freezing or stepwise freezing methods. However, the viability of seeds possessing elevated moisture content, such as *C. lansium*, *S. lychnophora*, and *S. nux-vomica*, exhibited diminished success after vitrification freezing and stepwise freezing in comparison to direct freezing. This outcome could potentially be attributed to the elevated concentration of cryoprotectants PVS2 or the prolonged duration of immersion in the freezing agent. Nevertheless, these suggestions remain speculative, necessitating further investigation to ascertain the true underlying causes.

The close association between the ultra-low temperature tolerance of plants and their ability to withstand dehydration, freezing at low temperatures, and vitrification has been well documented [38,39]. Seeds that possess normal or intermediate levels of dehydration tolerance can effectively eliminate free water prior to undergoing low-temperature freezing, resulting in a relatively broad spectrum of permissible moisture content. Consequently, the determination of the ideal moisture content, which varies across plant species, constitutes a pivotal factor in the preservation of seeds at ultra-low temperatures [33]. According to the Food and Agriculture Organization (FAO), the optimal moisture content for long-term storage of orthodox seeds falls within the range of 4 to 7%, a value range that aligns with the findings of this investigation. In the case of orthodox seeds, there is generally no notable disparity in viability pre- or post-freezing, provided the moisture content remains between 5 and 10%. This moisture range effectively prevents intracellular freezing damage resulting from excessive moisture at subzero temperatures, while also mitigating dehydration damage caused by insufficient moisture. The moisture content range deemed appropriate for intermediate seeds in this study predominantly fell within the range of 7 to 15%, whereas the suitable moisture content range for recalcitrant seeds lacked specificity.

### 3.2. The Roles of Seed Structure and Composition in the Cryopreservation Process

Seeds are subjected to significant physical pressure during the process of ultra-low temperature freezing and thawing. If the intercellular matrix of the seeds is unable to withstand this pressure, the seeds will suffer physical damage. This tolerance is closely associated with the moisture content of the seeds [34]. The seeds of *Albizia julibrissin* Durazz. [40] and *Euphorbia marginata* Pursh. [41] exhibited explosive behavior upon exposure to liquid nitrogen, whereas the robust seed shell of *Prunus pseudocerasus* (Lindl.) G. Don seeds [42] mitigated the immediate detrimental effects of temperature fluctuations on the seed coat. Consequently, seed coat rupture following freezing in liquid nitrogen is infrequent. The seed shell provides a protective shield, thereby minimizing the impact of moisture and serving a protective function. Within a specified range of water content, the present study demonstrated that recalcitrant seeds characterized by hard seed coats and thick kernels showed higher vitality after direct freezing in liquid nitrogen, while seeds with crisp or soft seed coats were best preserved through vitrification or stepwise freezing methods. It was initially hypothesized that the freezing of recalcitrant seeds at extremely low temperatures using liquid nitrogen may provide a protective effect due to the presence of a hard seed coat and a tightly packed seed kernel. Conversely, seeds possessing brittle or soft seed coats may necessitate the use of cryoprotectants to mitigate the potential adverse effects of liquid nitrogen freezing.

In conventional storage, seeds with high oil content often have shorter lifespans and are more difficult to store than other seeds containing carbohydrates or proteins. This is because fats are prone to rancidity that produces large amounts of toxic substances, resulting in a shortened lifespan of the seeds. In this study, seeds such as *C. anisum-olens*, *O. pinnata*, and *V. negundo* still had high activity after being frozen at a high moisture content, similar to the results of studies on *P. pseudocerasus* seeds [22] and *Crataegus pinnatifida* Bunge seeds [43]. Seeds rich in oil or those with a high lipid content have a higher tolerance to liquid nitrogen freezing at a higher moisture content. It may be that the high content of fats makes the seed’s free water less likely to freeze into ice, thereby reducing the damage of ice crystals to the seeds. This requires further investigation.

### 3.3. The Significance of Cryopreservation Technology and Cryobank for the Protection of Diversity of Medicinal Plant Genetic Resources

Presently, the genetic diversity of medicinal plants is confronted with numerous challenges [44], including environmental degradation due to anthropogenic activities and the depletion of resources resulting from over-harvesting and unsustainable utilization practices. These challenges have precipitated a sustained decline in the genetic diversity of medicinal plant resources, with certain species approaching the brink of extinction.

Each medicinal plant harbors distinct genetic information and possesses unique medicinal properties [45]. The preservation of these plants is crucial not only for maintaining biodiversity but also for safeguarding traditional medical knowledge. Currently, cryopreservation technology is instrumental in the conservation of plant genetic resources, offering advantages such as long-term stability and safety [46]. By employing cryopreservation and a cryobank, the viability of medicinal plant genetic material can be effectively prolonged, ensuring its enduring and stable conservation [47]. This is crucial for safeguarding the genetic resources of rare and endangered medicinal plants, thereby preserving their genetic integrity and diversity.

Cryopreservation technology, as an advanced method in the field, presents distinct advantages alongside certain limitations. Future research should focus on the development of novel cryoprotectants and optimized freezing protocols to enhance the efficacy of preservation and recovery of medicinal plants. Such advancements will not only broaden the applicability of preservation techniques but also offer more robust technical assurances for the long-term conservation of medicinal plant species.

## 4. Materials and Methods

### 4.1. Materials

The fruits of 164 medicinal plants (Table A1) were gathered in China. Subsequently, the peels and flesh were eliminated, and the seeds were extracted from the fruits. The unprocessed seeds, not subject to any dehydration procedures, were then enclosed in sealed bags and stored in a seed cabinet at a temperature of 10 °C for a maximum duration of 3 days. This was followed by an assessment of the initial moisture content and viability of the seeds. The experimental seeds were fresh seeds, all provided by the National Southern Medicine Gene Bank.

### 4.2. Storage Characteristic Determination Method

**Figure 5:**
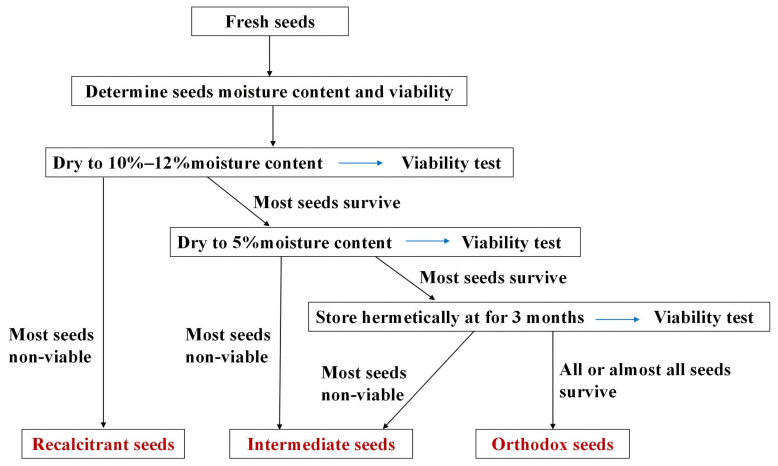
The steps for the determination of seed storage characteristics.

The method and steps (Figure 5) outlined by Hong et al. [48] were employed for the determination of seed storage characteristics. Subsequently, the experimental seed storage type was classified as orthodox, intermediate, or recalcitrant. The seeds were dehydrated to a 10–12% water content and their viability was checked. If most were non-viable, they were recalcitrant. If most were viable, they were further dehydrated to a 5% water content and checked again. If most were non-viable, they were intermediate. If most were viable, they were stored at −20 °C for 3 months and rechecked. If most were non-viable, they were intermediate; if nearly all were viable, they were orthodox seeds.

### 4.3. Determination of Seed Moisture Content and Viability

The seeds were enclosed in nylon mesh bags and positioned within a desiccator containing silica gel. Subsequently, the seeds were dried at room temperature for varying lengths of time based on their size and initial moisture content, thereby yielding seeds with distinct moisture content gradients. The high constant temperature drying method [49] was employed to ascertain the seeds’ moisture content (w). Specifically, three seeds were selected from refrigeration, their weight was recorded (m1), and they were then dehydrated in a drying oven set at 130 °C for 1 h before being reweighed (m2). The water content calculation formula was as follows: w = (m1 − m2)/m1 × 100%.

The 2,3,5-triphenyltetrazolium chloride (TTC) method [32] was used to assess seed viability before and after freezing. Fifteen seeds were peeled, halved along the ridge, placed in 1% TTC buffer (1 g TTC dissolved in every 100 mL of phosphate-buffer saline), incubated at 25 °C in the dark for 4 h, and then observed. Seeds with embryos and endosperms stained bright red and in normal condition were considered viable; others were deemed inactive.

### 4.4. Cryopreservation Experiments

A total of 164 varieties of medicinal plant seeds were exposed to ultra-low temperature freezing using liquid nitrogen. The viability of the seeds was assessed both before and after freezing, and the optimal freezing method for cryopreservation was determined for each seed. Each experimental seed underwent four treatments, including a control group and three freezing groups. The control group was not subject to freezing treatment, while the three freezing groups employed vitrification freezing, stepwise freezing, and direct freezing techniques, respectively [30].

Vitrification freezing: The cryopreservation tube containing the seeds was subjected to vitrification freezing using an MS solution consisting of 2.0 M glycerol and 0.4 M sucrose that served as the freezing protection loading solution. The tube was then kept at room temperature (25 ± 2 °C) for 20 min. Subsequently, the loading solution was substituted with PVS2 vitrification solution composed of 30% glycerol, 0.4 M sucrose, 15% ethylene glycol, and 15% dimethyl sulfoxide per liter of MS solution and placed in an ice bath for 30 min. Finally, the solution was rapidly replaced with pre-cooled fresh PVS2, and the tube was immediately transferred into liquid nitrogen for ultra-low temperature storage.

Stepwise freezing: The seeds designated for examination were immersed in cryotubes containing PVS2 (25 ± 2 °C) and subsequently stored in a refrigerator set at 4 °C for 30 min. Following this, the seeds were promptly transferred to a freezer at −20 °C for 1 h before being transferred into liquid nitrogen for preservation.

Direct freezing: The seeds to be stored were placed in the cryotube and quickly placed in liquid nitrogen for preservation.

Thawing: After 24 h, the seeds that had been subjected to freezing in liquid nitrogen were subsequently thawed at a temperature range of 37 to 40 °C in a water bath for 2–5 min. The seeds from both the stepwise freezing group and the vitrification freezing group were then subjected to three consecutive 5 min washes with MS solution containing 1.2 M sucrose, followed by two 10 min washes with sterile water

### 4.5. Physiological and Biochemical Indicators

Out of the species that were effectively frozen, a total of 62 species (40%) were chosen at random for the purpose of assessing the physiological and biochemical indicators of their seeds both prior to and following the freezing process. The seeds from both the control group and the optimal freezing group were ground in a freezing grinder to extract enzymes. The subsequent measurements included determining the relative conductivity following the ISTA protocol [49], quantifying the malondialdehyde (MDA) content using the thiobarbituric acid method, and assessing the catalase (CAT) activity through ultraviolet spectrophotometry. The guaiacol method was employed to determine the peroxidase (POD) activity, while the dehydrogenase activity and superoxide dismutase (SOD) activity were measured using the nitrogen blue tetrazolium (NBT) method. Additionally, the 3,5-dinitrosalicylic acid colorimetric method (DNS method) was used to determine the activity of α-amylase. The soluble protein content was determined using the Coomassie Brilliant Blue G250 method [50,51,52]. Due to the number of seeds, not all indicators were measured for some seeds.

### 4.6. Observation of Seed Internal Structure

From the effectively frozen species, 62 species were randomly chosen to observe the internal structure of these seeds before and after freezing. The seeds from both the control and optimally frozen groups were incised to expose the region containing the embryo, followed by paraffin sectioning. These seeds were immersed in FAA fixative [53] (formaldehyde: acetic acid: alcohol = 1:1:18) for 72 h at 4 °C. The fixed seeds were then dehydrated in ethanol solutions of 70%, 80%, 90%, 95%, and 100%, soaking for at least one hour in each. After dehydration, the seeds were cleared in xylene for at least 2 h, then immersed in molten paraffin at 56–58 °C for at least 4 h. The paraffin-impregnated seeds were transferred to pre-cooled molds and solidified at 4 °C into paraffin blocks. Using a Leica RM2245 semi-automatic rotary microtome, paraffin blocks were sliced into 8–12 micrometer sections. These sections were flattened on a 42 °C water surface, transferred to pre-warmed slides, and dried in a 37 °C incubator for at least 2 h. The dried sections were stained with Ponceau S for 1–2 h, rinsed with tap water, stained with Fast Green for 2 min, rinsed again, and air-dried. The stained sections were dehydrated in ethanol solutions (75%, 85%, 95%) for 5 min each, cleared in xylene for 5 min, and sealed with a neutral mounting medium and cover slip. The slides were then examined under an optical microscope to observe the seeds’ internal structure.

### 4.7. Statistical Analysis

SAS9.4 software was used for all statistical analysis. For all quantitative data, one-way ANOVA was used, followed by an LSD multiple range test when significant differences were detected (*p* < 0.05).

## 5. Conclusions

This study effectively employed cryopreservation to successfully preserve the seeds of 155 medicinal plants. By conducting a comprehensive analysis of moisture content, seed composition, and freezing methods, and employing microscopic observation as well as physiological and biochemical indicators, we identified consistent patterns for the cryopreservation of medicinal plant seeds. Specifically, our findings indicated the following. (1) The freezing method had minimal impact on over 90% of orthodox seeds, and the optimal freezing conditions involved a moisture content ranging from 5 to 10% and direct freezing. Notably, all seeds exhibited successful germination following the freezing process. (2) The freezing moisture content range of 7–15% was suitable for most intermediate seeds, and the direct freezing method was particularly appropriate for seeds with lower initial moisture content. (3) The direct freezing method is recommended for recalcitrant seeds characterized by a hard seed coat and a tightly packed seed kernel. Conversely, recalcitrant seeds that are brittle or soft are better suited for vitrification or stepwise freezing techniques. (4) The freezing of most seeds using liquid nitrogen did not yield any notable alterations in their appearance. (5) The application of cryopreservation adversely affects the biochemical markers of the majority of seeds, a phenomenon that was not significantly associated with the characteristics of seed storage. (6) Seeds exhibiting high recalcitrance exhibited diminished rates of germination after freezing, necessitating further investigation. The research establishes a scientific foundation and technical methodologies for the long-term preservation of medicinal plant germplasm resources, offers strategic responses for biodiversity conservation, and fosters the sustainable utilization of these resources.

## Figures and Tables

**Table 1:** Seed germination results after cryopreservation.

Species	Moisture Content	Freezing Methods	Viability		Germination after Freezing
			Control	Freezing	
*A. catechu*	35.24%	direct freezing	96.97%	96.97%	20%
*A. esculentus*	19.78%	direct freezing	100%	100%	33.33%
*A. heterophyllus*	46.53%	stepwise freezing	98.57%	76.19%	0
*A. sinensis*	10.12%	direct freezing	88.33%	89%	98.33%
*C. annuum*	30%	vitrification	80%	80%	80%
*C. argentea*	6.59%	stepwise freezing	40%	70%	40%
*C. chinensis*	37.69%	stepwise freezing	75%	47.78%	0
*C. halicacabum*	21.35%	vitrification	94.44%	70%	0
*C. junos*	24.35%	direct freezing	91.67%	98.33%	11.34%
*C. lacryma-jobi*	9.86%	direct freezing	85%	83.33%	10%
*C. lansium*	43%	direct freezing	100%	86.67%	0
*C. maritima*	9.32%	direct freezing	89.28%	95.24%	22.33%
*C. maxima*	7.03%	stepwise freezing	64.58%	68.12%	8.98%
*C. moschata*	6.02%	direct freezing	100%	95%	86.67%
*C. papaya*	16.94%	direct freezing	95%	95%	32%
*C. pulcherrima*	18.92%	stepwise freezing	80%	76.67%	90%
*C. reticulata*	23.65%	stepwise freezing	90%	90%	20%
*C. tinctorius*	8.40%	direct freezing	95%	95%	95.56%
*D. calycinum*	17%	vitrification	70%	50%	0
*D. indica*	24.69%	stepwise freezing	45%	38.33%	0
*D. longan*	14.42%	stepwise freezing	90%	80%	0
*F. indica*	17.03%	direct freezing	99.17%	99.17%	26.67%
*F. indica* Merr	5.21%	direct freezing	95%	95%	50%
*G. biloba*	19.77%	direct freezing	96.67%	98.33%	16.67%
*H. campechianum*	11.66%	vitrification	80%	84.17%	95.55%
*H. undatus*	10.49%	direct freezing	66.67%	71%	68.67%
*L. inermis*	27.27%	vitrification	76.67%	73.33%	60%
*M. argentea*	9.27%	direct freezing	65%	61.67%	9%
*M. citrifolia*	10.94%	direct freezing	75%	63.33%	0
*P. edulis*	10.25%	direct freezing	93.33%	80%	6.67%
*P. ginseng*	21.37%	direct freezing	95%	81.67%	6.67%
*P. granatum*	10.32%	stepwise freezing	78.33%	85%	10%
*P. indius*	8.61%	vitrification	84.31%	82.22%	66.67%
*P. nigrum*	21.77%	direct freezing	70%	24%	0
*P. perfoliatum*	15.06%	stepwise freezing	90%	92%	8.57%
*P. polyphylla*	51.99%	direct freezing	90%	90%	2%
*P. praeruptorum*	5.64%	vitrification	87.78%	93.33%	94.44%
*P. pseudoginseng*	38.31%	stepwise freezing	100%	63.33%	0
*P. vulgaris*	8.72%	direct freezing	100%	100%	75%
*R. communis*	5.35%	direct freezing	100%	95%	90%
*S. dulcificum*	27.83%	vitrification	100%	95.55%	13.67%
*S. lanceolata*	13.29%	direct freezing	100%	80%	45%
*S. lychnophora*	47.66%	direct freezing	100%	69.05%	0
*S. maritima*	31%	direct freezing	96.67%	50%	2%
*S. nux-vomica*	31.94%	direct freezing	92.22%	56.87%	40%
*S. sericea*	13.19%	direct freezing	73.33%	71.11%	1.67%
*T. candida*	14.44%	direct freezing	100%	100%	75%
*T. indica*	10.07%	direct freezing	95.24%	80.95%	85%
*Z. mays*	10.53%	direct freezing	100%	97.33%	97.78%
*Z. nitidum*	12.81%	direct freezing	85.19%	81.48%	17.89%

## Data Availability

The original contributions presented in the study are included in the article material, further inquiries can be directed to the corresponding author.

## Appendix A

**Table A1:** Storage characteristics and cryopreservation results of 164 medicinal plant seeds.

Families	Species	Initial Moisture Content	Freezing Moisture Content Range	Minimum Viability		Suitable Freezing Methods	Storage Characteristics
				Control	Freezing		
Amaranthaceae	*Celosia argentea* L. medicinal part: dry and mature seeds	≥24%	7–10%	26.67%	33.33%	stepwise freezing	lowly recalcitrant
Annonaceae	*Annona montana* Macf. medicinal part: fruit	≥22%	6–22%	100%	80%	stepwise freezing	intermediate
	*Annona reticulata* Linn. medicinal parts: bark, fruit, leaves	≥25%	5–10%	93.33%	93.33%	direct freezing	orthodox
	*Artabotrys hexapetalus* Medicinal part: root	≥15%	5–15%	96.5%	95.42%	direct freezing	orthodox
	*Polyalthia lauii* Merr. medicinal parts: roots and leaves	≥38%	25–28%	62.5%	75%	vitrification freezing	moderately recalcitrant
Apiaceae	*Peucedanum praeruptorum* Medicinal part: root	≥14%	5–10%	76.67%	85.55%	direct freezing	orthodox
Apocynaceae	*Rauvolfia vomitoria* medicinal parts: roots, stem bark	≥13%	11–14%	53.3%	60%	vitrification freezing	intermediate
Aquifoliaceae	*Ilex rotunda* Thunb. medicinal parts: leaves and bark	≥17%	6–11%	92.73%	87.76%	vitrification freezing	orthodox
Araceae	*Alocasia macrorrhiza* medicinal part: rootstock	≥45%	7–45%	76.67%	76.8%	direct freezing	intermediate
Araliaceae	*Panax pseudoginseng* Wall. var. notoginseng (Burkill) Hoo et Tseng medicinal part: rootstock	≥58%	38–48%	95%	90%	stepwise freezing	moderately recalcitrant
	*Panax ginseng* C. A. Mey. medicinal part: rootstock	≥30%	7–28%	75%	71.67%	direct freezing	intermediate
Arecaceae	*Areca catechu* L. medicinal part: seeds	≥57%	30–54%	80%	60%	direct freezing	highly recalcitrant
	*Arenga westerhoutii* Griffith medicinal part: fruit	≥30%	6–15%	16.67%	33.33%	vitrification freezing	moderately recalcitrant
	*Calamus tetradactylus Hance* medicinal part: whole plant	≥35%	10–25%	40%	80%	vitrification freezing	moderately recalcitrant
	*Trachycarpus fortunei* medicinal part: petiole	≥20%	8–15%	70%	60%	direct freezing	intermediate
Asclepiadaceae	*Telosma cordata* (Burm. f.) Merr. medicinal part: flower and leaves	≥17%	14–15%	80%	78.89%	vitrification freezing	intermediate
Asteraceae	*Arctium lappa* L. medicinal part: fruit	≥12%	6–12%	90%	93.33%	direct freezing	intermediate
	*Carthamus tinctorius* L. medicinal part: flower	≥10%	5–10%	94.44%	95%	direct freezing	orthodox
	*Xanthium strumarium* medicinal part: fruit	≥25%	5–15%	100%	100%	direct freezing	orthodox
Boraginaceae	*Messerschmidia argentea* (L. f.) Johnst. medicinal part: leaves and stem bark	≥20%	9–15%	64.17%	60.84%	direct freezing	orthodox
Cactaceae	*Hylocereus undatus* (Haw.) Britt. et Rose medicinal part: succulent stem and flower	≥15%	7–10%	61%	67%	direct freezing	intermediate
Cardiopteridaceae	*Gonocaryum lobbianum* (Miers) Kurz medicinal part: root	≥48%	40–45%	100%	50%	stepwise freezing	intermediate
Caricaceae	*Carica papaya* L. medicinal part: fruit and leaves	≥23%	6–17%	90%	90%	direct freezing	intermediate
Casuarinaceae	*Casuarina equisetifolia* Forst. medicinal part: branches and leaves	≥15%	7–15%	92%	93.3%	direct freezing	orthodox
Chenopodiaceae	*Atriplex repens* Roth medicinal part: whole herb	≥25%	12–16%	60%	55%	direct freezing	lowly recalcitrant
Cornaceae	*Cornus officinalis* Siebold & Zucc. medicinal part: ripe fruit	≥38%	25–28%	70%	66.67%	stepwise freezing	moderately recalcitrant
Cucurbitaceae	*Benincasa hispida* (Thunb.) Cogn. medicinal part: peel and seeds	≥35%	5–15%	60%	65%	direct freezing	orthodox
	*Cucurbita moschata* (Duch. ex Lam.) Duch. ex Poiret medicinal part: sarcocarp	≥24%	5–10%	96%	94%	direct freezing	orthodox
	*Luffa aegyptiaca* medicinal part: mature fruit vascular bundle, flower, melon stem	≥12%	5–12%	100%	100%	direct freezing	orthodox
	*Trichosanthes scabra* Loureiro medicinal part: whole herb	≥22%	10–15%	92%	90%	direct freezing	intermediate
Daphniphyllaceae	*Daphniphyllum calycinum* Benth. medicinal part: root and leaves	≥47%	30–33%	70%	40%	vitrification freezing	moderately recalcitrant
	*Daphniphyllum macropodium* Miq. medicinal part: seeds and leaves	≥13%	10–13%	100%	70%	stepwise freezing	intermediate
Dipterocarpaceae	*Vatica mangachapoi* Blanco medicinal part: fruit	≥25%	18–22%	53.33%	33.33%	direct freezing	highly recalcitrant
Ebenaceae	*Diospyros kaki* Thunb. medicinal part: persistent calyx	≥34%	7–15%	84%	96%	direct freezing	intermediate
	*Diospyros strigosa* Hemsl. medicinal part: fruit	≥45%	40–45%	90%	85%	stepwise freezing	moderately recalcitrant
Euphorbiaceae	*Hevea brasiliensis* medicinal part: leaves	≥30%	10–15%	100%	83.33%	direct freezing	intermediate
	*Mallotus philippensis* medicinal part: the powdery fuzz on the surface of fruit and root	≥15%	11–15%	26.67%	33.33%	stepwise freezing	lowly recalcitrant
	*Ricinus communis* L. medicinal part: seeds	≥12%	3–12%	100%	93.33%	direct freezing	orthodox
	*Trewia nudiflora* medicinal part: seeds	≥25%	5–10%	80%	83.33%	direct freezing	orthodox
Flagellariaceae	*Flagellaria indica* medicinal part: stems and rhizomes	≥24%	11–24%	94.17%	94%	direct freezing	intermediate
Ginkgoaceae	*Ginkgo biloba* L. medicinal part: leaves	≥43%	12–28%	73.68%	83.33%	direct freezing	intermediate
Goodeniaceae	*Scaevola sericea* Vahl medicinal part: leaves	≥17%	10–14%	61.96%	60%	direct freezing	orthodox
Guttiferae	*Cratoxylum cochinchinense* (Lour.) Bl. medicinal part: root, bark and tender leaves	≥11%	8–11%	76.67%	75%	vitrification freezing	intermediate
Icacinaceae	*Iodes vitiginea* (Hance) Hemsl. medicinal part: root	≥21%	18–20%	100%	80%	direct freezing	moderately recalcitrant
Lamiaceae	*Anisomeles indica* (Linnaeus) Kuntze medicinal part: whole herb	≥15%	5–10%	70%	40%	direct freezing	orthodox
	*Salvia hispanica* L. medicinal part: seeds	≥15%	5–10%	31%	92%	stepwise freezing	lowly recalcitrant
	*Vitex negundo* L. var. cannabifolia (Sieb. et Zucc.) Hand.-Mazz. medicinal part: leaves and seeds	≥30%	24–28%	44.94%	66.67%	direct freezing	lowly recalcitrant
	*Vitex quinata* (Lour.) Wall. medicinal part: root and trunk pith	≥16%	10–12%	88.33%	76.67%	vitrification freezing	lowly recalcitrant
	*Vitex rotundifolia* Linnaeus f. medicinal part: fruit	≥37%	6–10%	66.67%	60%	stepwise freezing	intermediate
	*Vitex trifolia* L medicinal part: fruit	≥15%	6–14%	90%	71.05%	vitrification freezing	orthodox
	*Vitex tripinnata* medicinal part: stem	≥20%	5–10%	40%	71.43%	vitrification freezing	lowly recalcitrant
Lauraceae	*Cinnamomum camphora* (L.) presl medicinal part: root, fruit, branch and leaves	≥25%	10–24%	90%	66.67%	direct freezing	intermediate
	*Cinnamomum burmannii*(Nees)Blume medicinal part: bark, root bark, leaves and branches	≥25%	5–10%	85%	85%	direct freezing	orthodox
	*Cinnamomum verum* medicinal part: bark	≥40%	33–37%	60%	33.33%	stepwise freezing	moderately recalcitrant
Leguminosae	*Abrus precatorius* medicinal part: root and vine	≥14%	6–14%	81.67%	61.9%	direct freezing	orthodox
	*Bauhinia purpurea* medicinal part: root, bark, flower and leaves	≥13%	5–10%	95.55%	95.55%	direct freezing	orthodox
	*Caesalpinia pulcherrima* (L.) Sw. medicinal part: root and stem bark	≥25%	15–19%	80%	76.67%	stepwise freezing	lowly recalcitrant
	*Canavalia maritima* (Aubl.) Thou. medicinal part: root	≥32%	6–32%	80%	67.98%	direct freezing	intermediate
	*Erythrophleum fordii* Oliv. medicinal part: seeds, bark	≥22%	10–20%	60%	90%	stepwise freezing	intermediate
	*Euchresta japonica* medicinal part: root, stem	≥30%	5–10%	100%	90%	direct freezing	orthodox
	*Haematoxylum campechianum* L. medicinal part: wood	≥20%	11–13%	56.25%	84.17%	vitrification freezing	lowly recalcitrant
	*Lablab purpureus* (Linn.) Sweet medicinal part: seeds	≥40%	5–12%	95%	95%	direct freezing	orthodox
	*Leucaena leucocephala* medicinal part: seeds	≥17%	5–10%	94.6%	93.33%	direct freezing	orthodox
	*Ormosia pinnata* (Lour.) Merr. medicinal part: wood	≥65%	45–60%	80.59%	81.84%	vitrification freezing	highly recalcitrant
	*Phaseolus vulgaris* Linn. medicinal part: seeds and whole herb	≥60%	7–10%	100%	95%	direct freezing	orthodox
	*Pongamia pinnata* medicinal part: whole plant	≥45%	15–42%	94.4%	87.38%	direct freezing	lowly recalcitrant
	*Senna tora (Linnaeus)* Roxburgh medicinal part: seeds	≥15%	5–15%	100%	100%	direct freezing	orthodox
	*Sesbania cannabina* medicinal part: leaves and seeds	≥18%	5–10%	100%	100%	direct freezing	orthodox
	*Sophora flavescens* Alt. medicinal part: root	≥10%	5–10%	100%	96.67%	direct freezing	orthodox
	*Tamarindus indica* Linn. medicinal part: fruit	≥20%	8–19%	83.33%	80.95%	direct freezing	orthodox
	*Tephrosia candida* medicinal part: leaves	≥15%	4–15%	100%	95%	direct freezing	orthodox
	*Cassia occidentalis* Linn. medicinal part: seeds, root and leaves	≥24%	4–24%	95%	95%	direct freezing	orthodox
	*Pterocarpus indius* medicinal part: heartwood	≥12%	8–11%	84.31%	88.89%	vitrification freezing	orthodox
	*Vigna marina* (Burm.) Merr. medicinal part: whole plant	≥30%	11–17%	85.83%	90%	direct freezing	intermediate
Liliaceae	*Cordyline fruticosa* (L.) A. Cheval. medicinal part: flower	≥35%	27–32%	86.67%	63.33%	vitrification freezing	intermediate
	*Paris polyphylla* medicinal part: rootstock	≥57%	13–52%	81.67%	63.33%	direct freezing	intermediate
	*Ophiopogon japonicus* (L. f.) Ker-Gawl. medicinal part: root	≥33%	15–20%	60%	90%	direct freezing	lowly recalcitrant
Loganiaceae	*Strychnos nux-vomica* L. medicinal part: seeds	≥32%	28–32%	97%	56%	direct freezing	moderately recalcitrant
Lythraceae	*Lawsonia inermis* L. medicinal part: bark	≥46%	27–43%	70%	72%	vitrification freezing	lowly recalcitrant
	*Lagerstroemia speciosa* (L.) Pers. medicinal part: root, leaves and seeds	≥15%	10–12%	48%	50%	vitrification freezing	intermediate
	*Punica granatum* L. medicinal part: leaves, root, stem bark and flower	≥15%	7–15%	88%	85%	stepwise freezing	intermediate
Malvaceae	*Abelmoschus esculentus* (L.) Moench medicinal part: root, leaves, flower and seeds	≥42%	7–20%	92%	94%	direct freezing	intermediate
	*Abroma augustum* (L.) L. f. medicinal part: root and leaves	≥25%	5–10%	96.67%	96.67%	direct freezing	orthodox
	*Abutilon indicum* medicinal part: whole herb and root	≥18%	5–10%	96.67%	100%	direct freezing	orthodox
	*Hibiscus mutabilis* L. medicinal part: flower, leaves and root	≥25%	18–25%	100%	90%	vitrification freezing	lowly recalcitrant
	*Hibiscus sabdariffa* L. medicinal part: calyx	≥20%	5–12%	88%	85%	direct freezing	orthodox
	*Sterculia lanceolata* Cav. medicinal part: root and leaves	≥30%	13–16%	46.67%	45%	direct freezing	moderately recalcitrant
	*Sterculia lychnophora* Hance medicinal part: seeds	≥70%	45–48%	95%	69.05%	direct freezing	highly recalcitrant
Meliaceae	*Melia azedarach* L. medicinal part: bark	≥17%	5–15%	86.67%	90%	direct freezing	orthodox
Menispermaceae	*Diploclisia glaucescens* Bl. medicinal part: root	≥45%	13–40%	75.55%	77.78%	direct freezing	lowly recalcitrant
Moraceae	*Antiaris toxicaria* Lesch. medicinal part: fresh tree juice and seeds	≥46%	24–32%	70%	73.33%	vitrification freezing	moderately recalcitrant
	*Artocarpus heterophyllus* Lam. medicinal part: fruit and kernel	≥58%	33–54%	75%	75%	stepwise freezing	highly recalcitrant
	*Malaisia scandens* (Lour.) Planch. medicinal part: root and leaves	≥45%	30–40%	85%	80%	vitrification freezing	moderately recalcitrant
Musaceae	*Musella lasiocarpa* (Franchet) C. Y. Wu ex H. W. Li medicinal part: flower	≥20%	12–20%	33.33%	53.33%	stepwise freezing	moderately recalcitrant
Myricaceae	*Morella rubra* medicinal part: bark	≥15%	5–15%	80%	80%	direct freezing	orthodox
Myrtaceae	*Syzygium aromaticum* (L.) Merr. & L. M. Perry medicinal part: flower bud	≥45%	19–45%	80%	0		highly recalcitrant
	*Syzygium bullockii* (Hance) Merr. et Perry medicinal part: fruit and root	≥48%	38–46%	60.42%	77.08%	stepwise freezing	lowly recalcitrant
	*Syzygium myrsinifolium* (Hance) Merr. et Perry medicinal part: fruit, flower, seeds and stem bark	≥48%	33–35%	33.33%	33.33%	stepwise freezing	highly recalcitrant
	*Psidium littorale* Raddi medicinal part: fruit	≥15%	7–12%	47.78%	52.22%	vitrification freezing	intermediate
	*Syzygium cumini* (L.) Skeels medicinal part: fruit	≥40%	22–40%				highly recalcitrant
	*Syzygium nervosum* Candolle medicinal part: flower, leaves and root	≥50%	10–50%				highly recalcitrant
Nelumbonaceae	*Nelumbo nucifera* medicinal part: stamens, receptacle, mature seeds and radicles	≥33%	5–10%	90%	93.3%	direct freezing	orthodox
Olacaceae	*Olax austrosinensis* Y. R. Ling medicinal part: root and leaves	≥21%	5–15%	80%	83.3%	vitrification freezing	intermediate
Oleaceae	*Ligustrum quihoui* Carr. medicinal part: root bark, leaves and fruit	≥16%	11–17%	70%	60%	direct freezing	intermediate
	*Osmanthus fragrans* Lour. medicinal part: flower, fruit and root	≥24%	8–15%	50%	90%	vitrification freezing	intermediate
Passifloraceae	*Passiflora foetida* L. medicinal part: leaves	≥28%	4–28%	95.42%	96.67%	direct freezing	orthodox
	*Passiflora edulis* Sims medicinal part: fruit	≥15%	10–15%	67%	78%	direct freezing	lowly recalcitrant
Phyllanthaceae	*Aporusa dioica* (Roxb.) Muell. Arg. medicinal part: leaves	≥43%	30–36%	60.8%	41.29%	vitrification freezing	moderately recalcitrant
	*Baccaurea ramiflora* Loureiro medicinal part: fruit	≥42%	30–35%	60%	46.67%	vitrification freezing	highly recalcitrant
	*Bischofia javanica* Bl. medicinal part: root	≥25%	14–18%	61.27%	50.28%	direct freezing	lowly recalcitrant
	*Bridelia balansae* Tutcher medicinal part: root	≥43%	6–43%				moderately recalcitrant
	Cleistanthus sumatranus (Miq.) Muell. Arg. medicinal part: leaves	≥20%	14–17%	42%	71%	vitrification freezing	intermediate
Piperaceae	*Piper nigrum* L. medicinal part: fruit	≥28%	21–22%	25%	23.33%		moderately recalcitrant
Pittosporaceae	*Pittosporum balansae* DC. medicinal part: root, leaves	≥45%	5–10%	50%	50%	direct freezing	orthodox
Poaceae	*Coix lacryma-jobi* L. medicinal part: kernel	≥12%	5–10%	81%	80%	direct freezing	orthodox
	*Zea mays* L. medicinal part: seeds and leaves	≥35%	5–12%	100%	96%	direct freezing	orthodox
Polygonaceae	*Polygonum perfoliatum* medicinal part: whole herb	≥23%	10–17%	88%	92%	stepwise freezing	intermediate
Primulaceae	*Ardisia crenata* Sims medicinal part: root and leaves	≥56%	48–52%	73.33%	90%	vitrification freezing	moderately recalcitrant
	*Ardisia humilis* Vahl medicinal part: bark	≥35%	7–15%	86.67%	86.67%	direct freezing	orthodox
	*Ardisia obtusa* Mez medicinal part: root	≥35%	19–32%	85%	87.3%	vitrification freezing	lowly recalcitrant
	*Embelia ribes* Burm. F. medicinal part: root and leaves	≥30%	18–20%	46.67%	33.33%	stepwise freezing	intermediate
	*Embelia scandens* (Lour.) Mez medicinal part: root and leaves	≥38%	35–38%	100%	90%	direct freezing	moderately recalcitrant
Putranjivaceae	*Drypetes indica* (Muell. Arg.) Pax et Hoffm. medicinal part: leaves	≥27%	20–25%	36%	38%	vitrification freezing	moderately recalcitrant
Ranunculaceae	*Coptis chinensis* Franch. medicinal part: rootstock	≥42%	30–38%	56.67%	47.78%	stepwise freezing	moderately recalcitrant
Rosaceae	*Eriobotrya japonica* (Thunb.) Lindl. medicinal part: leaves	≥55%	45–52%	90.61%	35.45%	vitrification freezing	highly recalcitrant
Rubiaceae	*Borreria articularis* (L. f.) G. Mey. medicinal part: whole herb	≥18%	7–18%	92.73%	67.19%	direct freezing	orthodox
	*Catunaregam spinosa* (Thunb.) Tirveng. medicinal part: root and leaves	≥15%	8–13%	95%	93.33%	direct freezing	orthodox
	*Coffea liberica* Bull ex Hiern medicinal part: seeds	≥40%	6–40%				highly recalcitrant
	*Morinda citrifolia* L. medicinal part: fruit, leaves, root, branch and trunk	≥20%	10–20%	71.67%	60%	direct freezing	lowly recalcitrant
Rutaceae	*Clausena anisum-olens* (Blanco) Merr. medicinal part: branch and leaves	≥55%	11–53%	61.67%	83.67%	stepwise freezing	lowly recalcitrant
	*Clausena excavata* medicinal part: root, leaves	≥44%	40–44%	100%	53.33%	vitrification freezing	moderately recalcitrant
	*Clausena lansium* (Lour.) Skeels medicinal part: root, leaves and seeds	≥45%	35–45%	81.7%	81.3%	direct freezing	highly recalcitrant
	*Citrus × junos* Siebold ex Tanaka medicinal part: seeds, peel	≥36%	7–25%	96%	93%	direct freezing	intermediate
	*Citrus maxima* (Burm.) Merr medicinal part: peel	≥20%	5–10%	93.33%	86.67%	direct freezing	orthodox
	*Citrus maxima* (Burm.) Merr. cv. ‘Tomentosay’ medicinal part: peel	≥15%	5–15%	60%	63.33%	stepwise freezing	intermediate
	*Citrus reticulata* Blanco medicinal part: peel	≥50%	10–30%	90%	82%	stepwise freezing	intermediate
	*Evodia lepta* medicinal part: root, leaves and fruit	≥24%	18–21%	52.33%	40.84%	direct freezing	moderately recalcitrant
	*Micromelum falcatum* (Lour.) Tan. medicinal part: root, leaves and root bark	≥35%	35–45%	60%	0		highly recalcitrant
	*Zanthoxylum bungeanum* Maxim. medicinal part: peel	≥20%	6–13%	80%	60%	direct freezing	intermediate
	*Zanthoxylum nitidum* (Roxb.) DC. medicinal part: root, stem, leaves and peel	≥18%	10–14%	66.67%	55.56%	direct freezing	intermediate
Salicaceae	*Flacourtia indica* (Burm. f.) Merr. medicinal part: fruit	≥21%	9–21%	75%	75%	direct freezing	orthodox
	*Hydnocarpus anthelminthica* medicinal part: seeds	≥25%	10–14%	87.8%	71.67%	stepwise freezing	intermediate
Santalaceae	*Santalum album* medicinal part: heartwood	≥20%	4–20%	91.11%	86.67%	direct freezing	orthodox
Sapindaceae	*Arytera littoralis* Bl. medicinal part: seeds	≥40%	26–28%	60%	71.43%	direct freezing	moderately recalcitrant
	*Cardiospermum halicacabum* L. medicinal part: whole plant	≥25%	15–25%	46.67%	55.93%	vitrification freezing	moderately recalcitrant
	*Dimocarpus longan* Lour. medicinal part: fruit	≥40%	12–25%	92%	80%	stepwise freezing	lowly recalcitrant
	*Litchi chinensis* Sonn. medicinal part: kernel	≥50%	43–48%	86.67%	66.67%	vitrification freezing	highly recalcitrant
Sapotaceae	*Synsepalum dulcificum* Daniell medicinal part: sarcocarp	≥40%	18–38%	84.12%	77.46%	vitrification freezing	moderately recalcitrant
Simaroubaceae	*Brucea javanica* (L.) Merr. medicinal part: seeds	≥17%	5–17%	82.22%	80%	direct freezing	orthodox
	*Suriana maritima* L. medicinal part: root and bark	≥39%	35–38%	96.67%	58.7%	direct freezing	moderately recalcitrant
Solanaceae	*Capsicum annuum* L. medicinal part: fruit	≥50%	30–45%	76%	74%	vitrification freezing	moderately recalcitrant
	*Datura metel* L. medicinal part: flower	≥42%	4–20%	93.33%	76.67%	direct freezing	orthodox
	*Solanum torvum* Swartz medicinal part: fruit	≥12%	7–10%	91.3%	87.76%	direct freezing	orthodox
Styracaceae	*Styrax suberifolius* medicinal part: root and leaves	≥45%	30–40%	60%	80%	direct freezing	lowly recalcitrant
	*Styrax tonkinensis* (Pierre) Craib ex Hartw. medicinal part: seed oil and resin	≥40%	24–35%	67.98%	25%		moderately recalcitrant
Thymelaeaceae	*Aquilaria sinensis* (Lour.) Spreng. medicinal part: resin	≥20%	10–16%	73.33%	73.33%	direct freezing	lowly recalcitrant
Vitaceae	*Vitis vinifera* L. medicinal part: fruit, rattan leaf and root	≥20%	5–10%	90.5%	70%	direct freezing	orthodox
Zingiberaceae	*Alpinia katsumadai* Hayata medicinal part: seeds	≥25%	12–16%	63.33%	56.67%	direct freezing	lowly recalcitrant
	*Alpinia maclurei* Merr. medicinal part: fruit	≥28%	15–25%	60%	50%	vitrification freezing	intermediate
	*Amomum muricarpum* Elm. medicinal part: fruit	≥20%	11–14%	60.99%	69.76%	stepwise freezing	lowly recalcitrant
	*Costus megalobractea* K. Schum. medicinal part: fruit	≥20%	13–16%	63.72%	60.97%	vitrification freezing	lowly recalcitrant
	*Hedychium coronarium* Koen. medicinal part: rootstock	≥78%	45–70%	61%	16%		highly recalcitrant
	*Zingiber corallinum* Hance medicinal part: rootstock	≥25%	8–24%	75.08%	73.33%	direct freezing	intermediate
	*Zingiber flavomaculosum* S.Q.Tong medicinal part: rootstock	≥20%	11–17%	84.82%	79.74%	direct freezing	lowly recalcitrant

**Table A2:** Changes in physiological and biochemical indicators of seeds before and after ultra-low temperature freezing.

Species	Test	REC (%)	MDA (μmol/g)	CAT (U/g)	POD (U/g)	SOD (U/g)	α-AA (U/g)	PRO (%)	TTCH (μg/mL)
*A. augustum*	A	9.21 ± 0.37 a	14.84 ± 0.63 a	138.17 ± 0.07 a	292.91 ± 0.62 a	3183.24 ± 0.39 a	60.39 ± 0.33 a	4.22 ± 0.07 a	19.44 ± 0.18 b
	B	5.50 ± 0.1 b	11.83 ± 0.57 b	92.64 ± 0.23 b	98.58 ± 0.31 b	3018.31 ± 0.42 b	60.23 ± 0.22 a	3.93 ± 0.17 b	20.64 ± 0.61 a
*A. catechu*	A	18.53 ± 0.01 b	6.81 ± 0.43 b	127.35 ± 0.47 a	1633.33 ± 7.02 b	701.32 ± 13.52 a	3.25 ± 1.91 a	3.63 ± 0.01 a	35.14 ± 0.49 a
	B	56.36 ± 0.02 a	10.83 ± 0.52 a	77.97 ± 0.01 b	5843.97 ± 17.74 a	587.59 ± 1.03 b	2.20 ± 1.92 a	3.23 ± 0.00 b	34.21 ± 1.47 a
*A. esculentus*	A	41.80 ± 0.45 b	14.33 ± 0.33 a	734.09 ± 0.51 a	107.09 ± 0.06 a	3166.75 ± 0.73 a	38.76 ± 0.38 b	5.65 ± 0.02 a	47.90 ± 0.53 a
	B	45.56 ± 0.9 a	13.90 ± 0.23 b	706.29 ± 0.18 b	44.68 ± 0.42 b	3067.7 ± 0.49 b	39.33 ± 0.46 a	5.64 ± 0.02 a	29.02 ± 0.16 b
*A. humilis*	A	4.58 ± 0.35 a	66.22 ± 1.62 b	160.14 ± 0.89 a	1789.17 ± 14.73 b	7.34 ± 0.27 b	121.88 ± 1.35 b	203.18 ± 0.5 b	66.65 ± 0.14 a
	B	3.70 ± 0.59 b	108.93 ± 0.05 a	86.52 ± 0.49 b	2337.20 ± 15.73 a	13.54 ± 0.30 a	134.37 ± 2.46 a	192.04 ± 0.41 a	62.57 ± 0.31 b
*A. heterophyllus*	A	5.33 ± 0.53 a	2.98 ± 0.5 b	104.88 ± 0.44 b	4083.69 ± 0.93 b	844.48 ± 0.74 a	23.27 ± 0.15 a	4.62 ± 0.42 a	18.93 ± 0.85 a
	B	4.60 ± 0.50 b	9.26 ± 0.47 a	143.53 ± 0.34 a	5845.39 ± 0.44 a	738.47 ± 0.20 b	23.02 ± 1.05 a	4.61 ± 0.09 a	3.79 ± 0.20 b
*A. katsumadai*	A	30.93 ± 0.76 a	0.93 ± 0.05 b	0.6 ± 0.03 a	515.66 ± 15.09 a	191.1 ± 4.68 a	4.08 ± 0.36 a	8.37 ± 2.25 b	
	B	31.98 ± 1.10 a	1.52 ± 0.49 a	0.62 ± 0.03 a	494.92 ± 4.27 b	178.31 ± 5.52 b	3.68 ± 0.20 b	39.74 ± 2.59 a	
*A. obtusa*	A	4.91 ± 0.46 b	50.97 ± 0.55 a	301.84 ± 3.63 b	1337.85 ± 1.68 a	6.56 ± 0.18 b	94.30 ± 0.43 b	27.40 ± 0.44 b	34.48 ± 0.21 a
	D	24.49 ± 0.24 a	36.10 ± 2.46 b	726.95 ± 3.71 a	1112.19 ± 7.13 b	14.04 ± 0.1 a	101.10 ± 0.51 a	32.02 ± 0.49 a	32.69 ± 0.67 b
*A. precatorius*	A	7.33 ± 0.28 a	313.00 ± 0.76 b	220.30 ± 0.44 a	7876.60 ± 3.38 a	3477.12 ± 0.98 a	30.22 ± 0.06 b	4.81 ± 1.08 a	63.64 ± 0.19 a
	B	1.14 ± 1.28 b	318.21 ± 1.56 a	101.37 ± 0.89 b	5306.38 ± 0.83 b	2642.33 ± 2.27 b	36.57 ± 0.15 a	5.21 ± 0.95 a	61.51 ± 0.28 b
*B. javanica*	A	65.41 ± 0.04 a	6.99 ± 0.6 a	73.45 ± 1.90 a	2078.58 ± 14.1 b	2306.17 ± 13.21 a	24.14 ± 1.25 a	4.31 ± 0.02 a	51.85 ± 1.40 a
	B	54.89 ± 0.06 b	7.17 ± 0.35 a	63.52 ± 1.00 b	2285.11 ± 17.44 a	2011.87 ± 11.41 b	23.76 ± 1.43 a	4.02 ± 0.02 b	25.43 ± 0.2 b
*A. sinensis*	A		1.26 ± 0.26 a	289.09 ± 12.04 a	52.55 ± 1.51 a	1650.68 ± 119.52 a	9.76 ± 1.00 b		2.13 ± 0.24 b
	B		1.36 ± 0.42 a	270.77 ± 8.75 b	44.64 ± 1.09 b	1826.52 ± 235.93 a	26.80 ± 1.14 a		2.64 ± 0.13 a
*C. annuum*	A	66.49 ± 1.65 b	24.23 ± 0.74 b	157.8 ± 1.93 b	1431.21 ± 1.75 b	1551.42 ± 1.91 b	13.73 ± 1.05 a	4.49 ± 0.09 b	4.63 ± 0.52 b
	D	69.76 ± 0.17 a	30.88 ± 1.69 a	541.13 ± 1.02 a	2850.35 ± 1.74 a	2061.17 ± 1.80 a	6.57 ± 0.60 b	4.93 ± 0.15 a	7.61 ± 1.00 a
*C. burmannii*	A	11.70 ± 1.36 b	1.57 ± 0.87 a	431.96 ± 0.97 b	8082.27 ± 7.32 a	3245.58 ± 2.88 a	14.97 ± 0.49 b	2.87 ± 0.29 a	46.49 ± 0.17 b
	B	14.77 ± 0.51 a	1.08 ± 0.51 a	506.03 ± 1.74 a	981.56 ± 1.09 b	2524.34 ± 3.45 b	15.74 ± 0.35 a	3.15 ± 0.28 a	55.94 ± 0.87 a
*C. camphora*	A		3.65 ± 0.88 b	28.81 ± 1.54 b	614.89 ± 36.56 b	2609.93 ± 20.14 a	22.96 ± 2.88 a	267.83 ± 2.27 a	76.78 ± 1.13 a
	B		6.34 ± 2.15 a	40.60 ± 2.55 a	1306.95 ± 78.26 a	2638.30 ± 39.16 a	21.00 ± 0.49 a	266.20 ± 14.57 a	44.12 ± 1.04 b
*C. fruticosa*	A	16.59 ± 0.16 b	7.38 ± 0.54 a	476.42 ± 0.23 a	10597.16 ± 0.13 a	2535.84 ± 1.44 b	43.27 ± 0.2 a	3.78 ± 0.38 a	6.51 ± 0.53 a
	D	25.05 ± 0.39 a	6.25 ± 0.41 b	456.65 ± 0.29 b	9823.40 ± 0.25 b	3010.36 ± 0.56 a	40.91 ± 0.37 b	4.01 ± 0.62 a	5.47 ± 0.09 b
*C. junos*	A	6.92 ± 0.5 b	11.42 ± 0.62 b	141.09 ± 1.92 a	10,393.62 ± 14.08 a	1319.54 ± 5.05 a	10.54 ± 1.47 b	5.16 ± 0.14 a	34.86 ± 0.71 a
	B	15.08 ± 1.81 a	15.34 ± 0.92 a	92.86 ± 2.62 b	10,170.21 ± 28.24 b	929.37 ± 3.59 b	13.54 ± 0.33 a	4.59 ± 0.25 b	24.10 ± 0.19 b
*C.lacryma-jobi*	A	36.84 ± 1.02 b	4.84 ± 0.43 a	118.35 ± 1.81 b	10,287.94 ± 13.59 a	2600.47 ± 3.78 b	12.16 ± 1.27 a	2.91 ± 0.20 a	15.96 ± 0.71 b
	B	49.07 ± 1.55 a	4.00 ± 0.69 b	245.21 ± 1.58 a	10,178.01 ± 16.42 b	2718.68 ± 5.52 a	12.29 ± 1.45 a	2.83 ± 0.19 a	17.77 ± 0.84 a
*C. lansium*	A		1.02 ± 0.26 a	55.04 ± 2.91 a	90,184.78 ± 28.31 b	87,213.60 ± 34.12 a	14.04 ± 0.56 b		25.01 ± 1.29 a
	B		0.97 ± 0.23 a	50.19 ± 0.85 b	118,081.04 ± 23.82 a	61,162.08 ± 56.25 b	17.51 ± 0.54 a		11.09 ± 1.91 b
*C. maritima*	A	76.59 ± 0.82 a	10.11 ± 0.62 a	599.29 ± 6.14 a	9155.32 ± 20.48 a	2502.01 ± 4.56 a	66.67 ± 0.75 b	6.28 ± 0.51 a	130.43 ± 1.33 a
	B	53.53 ± 1.34 b	9.17 ± 1.19 a	88.57 ± 5.47 b	5572.34 ± 18.30 b	1222.57 ± 12.12 b	67.97 ± 0.61 a	6.18 ± 0.26 a	94.15 ± 1.22 b
*C. maxima*	A	53.00 ± 0.40 a	7.2 ± 0.2 a	349.87 ± 0.23 b	2590.07 ± 0.63 b	2941.48 ± 0.08 a	46.02 ± 1.42 b	5.77 ± 0.31 b	61.61 ± 0.23 a
	C	35.43 ± 0.66 b	5.14 ± 1.07 b	520.61 ± 0.24 a	3175.89 ± 1.1 a	2040.92 ± 0.56 b	49.04 ± 1.96 a	6.53 ± 0.09 a	26.23 ± 0.28 b
*C.moschata*	A	40.45 ± 2.07 a	2.23 ± 0.66 a	425.75 ± 1.62 b	18,075.89 ± 19.84 b	3002.36 ± 5.96 a	18.86 ± 1.03 a	4.15 ± 0.29 a	36.00 ± 0.68 b
	B	28.34 ± 0.65 b	2.61 ± 0.89 a	507.98 ± 0.36 a	19,784.40 ± 34.15 a	2765.96 ± 9.76 b	14.58 ± 2.63 b	3.71 ± 0.15 b	37.54 ± 0.08 a
*C. occidentalis*	A	14.81 ± 1.11 b	28.15 ± 0.32 a	93.39 ± 0.40 a	8074.47 ± 0.06 b	3260.06 ± 0.48 a	43.28 ± 0.23 a	5.93 ± 0.43 b	235.77 ± 0.38 a
	B	17.66 ± 1.26 a	25.59 ± 0.33 b	80.63 ± 0.36 b	8582.98 ± 0.63 a	3111.16 ± 0.61 b	34.44 ± 0.44 b	6.38 ± 0.23 a	209.78 ± 0.39 b
*C. papaya*	A	54.44 ± 0.06 a	13.41 ± 0.58 a	55.23 ± 0.69 b	11498.58 ± 2.04 a	2474.62 ± 2.17 a	38.48 ± 0.47 a	1.01 ± 0.32 a	62.08 ± 0.49 b
	B	55.04 ± 0.47 b	14.82 ± 1.74 a	225.14 ± 0.68 a	10325.53 ± 0.62 b	2359.81 ± 2.02 b	29.94 ± 0.63 b	0.87 ± 0.18 a	70.47 ± 0.17 a
*C. pulcherrima*	A	33.06 ± 2.44 a	11.92 ± 0.84 a	1201.82 ± 16.07 a	8709.93 ± 3.28 a	2228.47 ± 19.35 a	6.19 ± 1.13 b	5.05 ± 0.4 a	127.88 ± 0.17 b
	C	29.78 ± 0.84 a	12.61 ± 1.61 a	82.05 ± 0.32 b	3862.41 ± 2.01 b	2062.22 ± 16.03 b	9.08 ± 0.67 a	5.01 ± 0.67 a	137.84 ± 0.37 a
*C. reticulata*	A	10.17 ± 1.07 b	11.70 ± 0.13 a	340.47 ± 0.68 b	3734.04 ± 2.71 b	2482.27 ± 4.4 a	5.89 ± 0.54 a	3.45 ± 0.22 a	72.71 ± 0.54 b
	C	28.29 ± 0.51 a	11.92 ± 1.00 a	345.26 ± 0.73 a	7443.26 ± 2.46 a	2423.17 ± 6.72 b	4.88 ± 1.03 a	3.68 ± 0.22 a	115.77 ± 2.68 a
*C.tinctorius*	A	50.39 ± 1.06 a	3.74 ± 2.01 a	651.42 ± 2.02 a	3139.01 ± 13.59 b	761.75 ± 3.28 b	11.39 ± 0.85 b	4.41 ± 0.07 a	9.93 ± 0.99 a
	B	44.98 ± 1.00 b	5.33 ± 0.91 a	425.53 ± 3.90 b	5265.96 ± 28.74 a	2180.19 ± 10.44 a	13.93 ± 0.82 a	4.37 ± 0.09 a	8.79 ± 0.29 b
*D. calycinum*	A	16.09 ± 0.06 a	9.88 ± 0.82 a	222.38 ± 1.49 b	2250.71 ± 23.51 b	472.81 ± 0.13 b	13.15 ± 0.61 a	237.44 ± 0.81 b	28.79 ± 1.72 a
	D	11.98 ± 0.02 b	8.40 ± 0.50 b	325.89 ± 0.8 a	2368.79 ± 33.44 a	945.63 ± 2.47 a	2.33 ± 0.44 b	261.36 ± 0.79 a	27.58 ± 0.22 a
*D. kaki*	A	54.38 ± 0.8 a	9.03 ± 1.27 a	657.00 ± 2.29 a	902.84 ± 2.00 b	2634.25 ± 3.73 b	6.41 ± 1.15 a	2.64 ± 0.40 a	27.86 ± 2.14 a
	B	53.76 ± 0.96 a	8.91 ± 1.13 a	419.55 ± 1.47 b	1678.72 ± 5.94 a	3647.42 ± 6.45 a	5.52 ± 0.87 a	2.25 ± 0.12 a	9.84 ± 1.70 b
*D. longan*	A	19.18 ± 1.82 b	30.87 ± 0.73 a	511.39 ± 0.66 b	2324.82 ± 3.26 a	3444.78 ± 4.48 a	25.06 ± 0.43 a	2.35 ± 0.46 a	63.32 ± 0.72 a
	C	22.15 ± 1.13 a	23.39 ± 0.88 b	688.61 ± 1.19 a	944.68 ± 0.91 b	2228.98 ± 7.45 b	16.15 ± 0.49 b	2.44 ± 0.14 a	60.12 ± 0.31 b
*D. metel*	A	14.47 ± 1.43 b	8.14 ± 1.41 a	227.35 ± 0.77 a	8853.90 ± 29.6 a	606.55 ± 2.61 a	27.97 ± 0.33 a	5.09 ± 0.33 b	6.11 ± 1.04 a
	B	21.9 ± 6.26 a	9.13 ± 0.43 a	145.30 ± 0.83 b	8801.42 ± 61.67 a	483.34 ± 1.12 b	21.30 ± 0.36 b	5.47 ± 0.21 a	3.89 ± 0.68 b
*E. lepta*	A	49.8 ± 0.57 a	24.78 ± 2.22 a	16.41 ± 0.77 a	6.15 ± 1.4 a	25.26 ± 2.01 b	8.69 ± 1.13 a	112.62 ± 11.78 a	39.84 ± 2.41 b
	B	47.68 ± 2.01 b	20.81 ± 2.34 b	16.44 ± 0.80 a	1.13 ± 0.03 b	58.4 ± 2.27 a	7.99 ± 1.19 a	101.83 ± 21.97 a	48.7 ± 2.56 a
*F. indica*	A	70.52 ± 0.24 a	14.85 ± 0.98 b	84.22 ± 1.44 b	1136.17 ± 0.42 a	263.22 ± 1.21 b	11.57 ± 0.25 b	4.23 ± 0.21 a	127.44 ± 0.4 b
	B	70.05 ± 0.45 b	18.27 ± 0.11 a	140.38 ± 0.21 a	852.48 ± 0.12 b	405.79 ± 1.72 a	19.06 ± 0.47 a	3.75 ± 0.18 b	151.28 ± 0.73 a
*G. biloba*	A	56.65 ± 1.75 a	3.78 ± 0.46 a	1667.28 ± 2.86 a	2309.93 ± 1.28 a	1105.90 ± 1.76 b	27.57 ± 1.94 a	2.72 ± 0.05 a	188.58 ± 2.42 a
	B	42.07 ± 1.92 b	4.32 ± 0.99 a	811.75 ± 1.16 b	914.18 ± 0.88 b	1682.89 ± 5.14 a	26.39 ± 1.37 a	2.59 ± 0.16 a	110.47 ± 1.13 b
*H. anthelminthica*	A		5.49 ± 1.33 a	12.47 ± 0.19 b	520.57 ± 21.90 b	2581.56 ± 46.27 a	24.76 ± 0.81 a	166.59 ± 3.75 a	4.38 ± 1.03 a
	C		4.12 ± 1.34 b	19.88 ± 2.84 a	814.61 ± 59.967 a	2170.21 ± 52.12 b	16.31 ± 1.24 b	164.30 ± 2.76 a	2.69 ± 0.45 b
*H. mutabilis*	A	12.48 ± 1.83 a	10.17 ± 1.39 a	655.80 ± 0.89 b	3631.21 ± 4.25 b	840.56 ± 3.74 b	19.78 ± 0.53 a	4.62 ± 0.04 a	6.26 ± 0.22 a
	D	7.15 ± 0.38 b	8.08 ± 1.72 b	821.81 ± 0.95 a	4304.96 ± 3.86 a	1523.51 ± 5.91 a	13.56 ± 0.42 b	4.18 ± 0.2 b	6.34 ± 1.14 a
*H.sabdariffa*	A	23.24 ± 0.57 b	34.20 ± 0.23 a	401.77 ± 2.67 b	10283.69 ± 3.11 a	1418.44 ± 6.02 b	26.67 ± 1.98 a	5.24 ± 0.33 a	46.74 ± 0.12 a
	B	28.45 ± 1.06 a	25.46 ± 0.86 b	917.82 ± 0.77 a	4097.87 ± 3.66 b	1442.08 ± 3.27 a	23.42 ± 0.78 b	4.86 ± 0.69 a	39.55 ± 0.43 b
*H. undatus*	A	20.85 ± 1.14 a	34.12 ± 1.11 a	791.71 ± 1.4 b	6446.81 ± 3.01 a	1013.17 ± 2.4 a	21.86 ± 1.35 a	3.67 ± 0.21 b	4.63 ± 0.22 a
	B	21.78 ± 2.02 a	29.76 ± 0.72 b	795.43 ± 1.95 a	1133.33 ± 1.39 b	607.90 ± 1.71 b	13.60 ± 0.22 b	4.35 ± 0.30 a	4.63 ± 0.24 a
*L. purpureus*	A	8.62 ± 1.19 b	143.35 ± 2.54 b	286.93 ± 4.11 b	13,351.06 ± 36.77 b	3711.58 ± 7.41 a	34.96 ± 1.00 a	3.94 ± 0.16 a	177.86 ± 0.60 a
	B	19.90 ± 0.90 a	194.34 ± 0.33 a	501.51 ± 5.88 a	4795.04 ± 12.32 a	3167.85 ± 7.73 b	31.92 ± 0.82 b	3.85 ± 0.29 a	172.33 ± 2.07 b
*M. argentea*	A	44.29 ± 0.81 a	10.89 ± 0.76 a	1.64 ± 1.01 b	8414.18 ± 11.83 b	1648.99 ± 9.94 a	30.56 ± 0.41 a	3.56 ± 0.38 a	
	B	39.63 ± 0.72 b	9.10 ± 1.37 b	13.56 ± 1.42 a	9006.38 ± 67.71 a	1113.65 ± 18.24 b	25.50 ± 0.26 b	3.81 ± 0.43 a	
*M. citrifolia*	A	61.87 ± 0.51 b	9.44 ± 1.10 b	54.74 ± 2.82 a	7770.92 ± 66.5 b	947.73 ± 22.98 b	3.98 ± 0.14 a	4.23 ± 0.1 a	2.96 ± 0.46 a
	B	64.54 ± 0.19 a	11.23 ± 1.10 a	9.22 ± 0.53 b	10,114.18 ± 47.65 a	1197.30 ± 7.38 a	3.98 ± 0.34 a	3.90 ± 0.15 b	2.77 ± 0.12 a
*O. pinnata*	A	14.47 ± 1.43 b	32.86 ± 0.38 b	157.98 ± 2.10 a	3577.30 ± 0.29 b	2836.88 ± 0.08 a	52.21 ± 0.35 a	4.77 ± 1.31 a	34.06 ± 1.38 a
	D	21.90 ± 1.26 a	60.57 ± 0.54 a	80.27 ± 0.10 b	4129.79 ± 0.09 a	2127.66 ± 0.14 b	52.54 ± 1.24 a	4.62 ± 0.78 a	18.53 ± 0.37 b
*P. edulis*	A	31.12 ± 1.24 a	12.87 ± 1.17 a	316.18 ± 1.63 a	8925.53 ± 5.13 a	1891.25 ± 5.22 b	5.32 ± 0.23 a	1.85 ± 0.04 a	7.56 ± 0.54 a
	B	34.02 ± 0.46 b	12.67 ± 1.36 a	255.01 ± 1.32 b	6415.60 ± 4.62 b	1950.35 ± 0.00 a	5.81 ± 0.73 a	1.97 ± 0.25 a	7.90 ± 0.9 a
*P. ginseng*	A	39.94 ± 0.38 b	7.74 ± 0.01 a	106.78 ± 0.58 b	9951.06 ± 0.36 b	2780.75 ± 1.00 b	40.29 ± 0.53 a	6.39 ± 1.08 a	3.49 ± 0.54 a
	B	46.66 ± 0.24 a	6.47 ± 0.45 b	291.75 ± 0.29 a	10524.11 ± 0.72 a	2824.12 ± 1.54 a	35.53 ± 0.24 b	7.18 ± 0.61 a	3.53 ± 0.48 a
*P. granatum*	A	49.40 ± 0.86 a	39.50 ± 1.26 b	266.04 ± 1.10 a	10041.84 ± 9.64 a	1058.75 ± 6.19 b	39.87 ± 1.07 a	2.64 ± 0.1 a	4.02 ± 1.24 b
	C	35.00 ± 2.63 b	42.69 ± 0.32 a	90.60 ± 1.81 b	6217.73 ± 0.86 b	1692.78 ± 5.55 a	38.77 ± 1.34 a	2.42 ± 0.27 a	5.83 ± 0.27 a
*P. indius*	A	5.59 ± 0.60 a	26.93 ± 0.40 a	84 ± 0.41 b	8622.70 ± 0.27 a	3803.76 ± 2.51 b	47.81 ± 0.20 a	6.02 ± 0.16 a	107.73 ± 0.25 b
	D	6.17 ± 0.39 a	22.1 ± 0.13 b	135.15 ± 0.43 a	7557.45 ± 0.28 b	3819.69 ± 2.55 a	46.22 ± 0.82 b	5.36 ± 0.23 b	141.28 ± 0.14 a
*P. perfoliatum*	A	49.77 ± 1.16 a	7.72 ± 0.87 b	375.53 ± 3.86 b	303.55 ± 3.9 b	1418.44 ± 1.51 b	10.85 ± 0.73 a	1.45 ± 0.04 a	8.30 ± 1.07 b
	C	42.24 ± 0.55 b	11.30 ± 0.44 a	634.97 ± 4.83 a	1987.23 ± 2.11 a	2765.96 ± 2.90 a	5.73 ± 0.66 b	1.30 ± 0.11 b	9.82 ± 1.38 a
*P. pinnata*	A	19.5 ± 0.03 b	34.28 ± 1.95 a	86.61 ± 0.10 b	2529.36 ± 12.12 a	2576.35 ± 14.77 b	27.62 ± 1.94 b	264.63 ± 3.09 a	37.01 ± 0.63 a
	B	34.88 ± 0.00 a	35.14 ± 2.35 a	125.09 ± 0.82 a	2293.33 ± 22.07 b	2991.27 ± 10.18 a	32.20 ± 1.94 a	255.41 ± 3.46 b	33.53 ± 1.44 b
*P. polyphylla*	A	6.73 ± 1.65 b	9.65 ± 0.35 a	100.22 ± 0.65 b	16023.40 ± 2.45 a	2023.31 ± 3.70 a	7.35 ± 0.49 b	4.09 ± 0.54 a	25.88 ± 0.58 a
	B	40.67 ± 0.13 a	5.41 ± 0.27 b	103.76 ± 0.53 a	13856.03 ± 4.99 b	1142.53 ± 2.69 b	8.34 ± 0.08 a	3.35 ± 0.54 b	14.86 ± 1.23 b
*P. pseudoginseng*	A	2.73 ± 0.26 b	4.66 ± 0.6 b	774.20 ± 18.15 a	68,913.48 ± 396.26 a	765.35 ± 17.62 a	22.68 ± 0.64 a	6.79 ± 0.23 b	13.54 ± 0.23 b
	C	5.96 ± 0.28 a	6.49 ± 1.15 a	605.54 ± 33.72 b	60,415.60 ± 390.11 b	510.23 ± 23.15 b	20.14 ± 0.22 b	7.04 ± 0.52 a	39.99 ± 0.54 a
*P. vulgaris*	A	41.80 ± 0.45 b	237.54 ± 1.13 a	340.92 ± 1.52 a	5268.09 ± 8.10 a	2193.33 ± 6.17 b	15.06 ± 0.97 b	4.52 ± 0.07 a	47.90 ± 0.53 a
	B	45.56 ± 0.90 a	177.71 ± 0.47 b	249.60 ± 0.96 b	5142.55 ± 3.01 b	2324.67 ± 7.26 a	21.27 ± 0.45 a	4.65 ± 0.19 a	29.02 ± 0.16 b
*R. communis*	A	46.3 ± 0.02 b	2.83 ± 0.65 a	541.62 ± 1.22 b	5712.77 ± 12.16 b	679.21 ± 0.84 b	9.67 ± 0.52 a	4.66 ± 0.00 b	78.47 ± 1.17 a
	B	49.03 ± 0.01 a	1.93 ± 0.57 b	610.20 ± 0.76 a	9577.30 ± 14.77 a	830.85 ± 0.62 a	8.94 ± 0.17 b	5.09 ± 0.00 a	76.59 ± 1.18 b
*S. album*	A	15.17 ± 1.37 b	2.10 ± 0.54 a	122.56 ± 1.31 b	819.86 ± 1.98 b	2868.64 ± 20.13 a	51.16 ± 2.46 b	1.70 ± 0.23 a	51.76 ± 2.6 a
	B	17.49 ± 1.85 a	2.70 ± 0.65 a	181.69 ± 2.29 a	1013.48 ± 8.54 a	2709.85 ± 8.46 b	56.40 ± 0.81 a	1.84 ± 0.11 a	50.81 ± 2.46 a
*S. dulcificum*	A		5.19 ± 0.53 b	2.20 ± 0.20 b	1302.29 ± 5.80 b	1919.86 ± 12.19 a	53.11 ± 2.91 a		4.15 ± 0.07 a
	D		7.38 ± 0.39 a	31.72 ± 1.79 a	1445.26 ± 37.26 a	1871.99 ± 15.94 b	48.71 ± 1.78 b		0.91 ± 0.03 b
*S. flavescens*	A	4.08 ± 1.71 a	57.05 ± 0.66 b	495.26 ± 1.43 a	10,315.60 ± 27.49 b	2771.21 ± 3.52 a	9.66 ± 0.20 b	5.45 ± 0.17 a	33.30 ± 0.80 a
	B	3.5 ± 0.84 a	64.43 ± 1.95 a	259.84 ± 1.23 b	8539.72 ± 22.34 a	2758.08 ± 2.72 b	15.43 ± 1.42 a	5.54 ± 0.12 a	24.12 ± 1.31 b
*S. hispanica*	A	25.99 ± 1.87 a	17.86 ± 1.07 b	567.91 ± 2.43 a	5509.93 ± 15.68 b	530.39 ± 0.62 b	22.35 ± 0.66 a	4.04 ± 0.29 a	4.44 ± 0.6 a
	C	25.9 ± 1.45 a	26.92 ± 1.06 a	195.26 ± 2.70 b	10441.13 ± 7.13 a	1396.09 ± 6.01 a	24.09 ± 1.83 a	3.63 ± 0.14 b	3.37 ± 0.16 b
*S. lanceolata*	A	17.42 ± 0.05 b	12.07 ± 0.43 b	200.04 ± 0.69 b	2064.68 ± 22.44 a	2706.61 ± 8.36 a	50.14 ± 0.49 b	246.39 ± 2.73 a	47.16 ± 0.89 a
	B	72.42 ± 0.39 a	13.57 ± 0.22 a	254.48 ± 0.94 a	2033.05 ± 25.73 b	2687.32 ± 40.72 a	51.90 ± 0.38 a	182.99 ± 2.68 b	47.90 ± 0.70 a
*S. lychnophora*	A	14.47 ± 0.01 b	6.33 ± 1.12 a	953.81 ± 22.79 a	1514.89 ± 35.51 b	861.03 ± 8.84 b	13.33 ± 0.79 a	3.96 ± 0.00 b	34.06 ± 1.38 a
	B	21.90 ± 0.06 a	5.95 ± 0.47 a	952.97 ± 12.16 a	7319.15 ± 25.59 a	1314.21 ± 22.41 a	10.05 ± 1.47 b	4.39 ± 0.00 a	18.53 ± 0.37 b
*S. maritima*	A	65.40 ± 1.58 b	29.23 ± 0.94 b	1077.08 ± 3.74 a	436.17 ± 3.91 b	726.59 ± 4.27 b	33.66 ± 0.30 a	4.29 ± 0.21 a	
	B	70.42 ± 1.09 a	32.77 ± 0.59 a	305.19 ± 0.61 b	493.62 ± 6.77 a	1023.55 ± 4.07 a	25.20 ± 0.25 b	3.85 ± 0.12 b	
*S. nux-vomica*	A	25.06 ± 4.96 b	3.94 ± 1.39 a	1125.31 ± 36.51 a	1312.06 ± 16.51 b	1459.26 ± 30.3 a	16.62 ± 0.14 b	4.16 ± 0.25 a	18.03 ± 1.75 a
	B	31.34 ± 4.01 a	2.19 ± 0.96 b	773.80 ± 24.17 b	1917.73 ± 56.52 a	887.80 ± 14.47 b	19.16 ± 0.35 a	4.99 ± 1.4 a	2.31 ± 0.19 b
*S. sericea*	A	80.6 ± 0.48 b	10.17 ± 0.71 a	649.42 ± 3.82 a	8649.65 ± 32.33 b	145.32 ± 1.93 b	5.69 ± 0.48 b	3.11 ± 0.12 a	4.76 ± 0.38 a
	B	82.90 ± 0.72 a	10.58 ± 0.56 a	67.73 ± 2.4 b	8731.91 ± 16.57 a	852.96 ± 3.56 a	11.14 ± 0.72 a	2.94 ± 0.09 b	3.87 ± 0.34 b
*T. candida*	A	2.67 ± 0.58 a	1.61 ± 0.51 b	1057.23 ± 31.06 a	41,707.80 ± 324.87 a	2714.42 ± 52.45 a	38.67 ± 1.76 a	7.76 ± 0.84 a	74.00 ± 2.12 a
	B	2.95 ± 0.46 a	5.97 ± 2.22 a	883.20 ± 30.22 b	41,831.91 ± 326.50 a	2296.04 ± 54.62 b	40.06 ± 0.45 a	7.29 ± 0.23 a	56.70 ± 0.32 b
*T. scabra*	A	33.74 ± 2.18 b	3.39 ± 1.13 a	109.75 ± 2.53 a	909.22 ± 23.71 b	3054.94 ± 16.77 a	24.60 ± 0.14 b	2.99 ± 0.19 a	10.73 ± 1.44 a
	B	40.77 ± 0.15 a	2.16 ± 0.15 b	13.70 ± 0.11 b	2126.95 ± 14.96 a	2127.66 ± 4.53 b	27.06 ± 0.23 a	3.11 ± 0.12 a	8.68 ± 1.45 b
*V. marina*	A		60.65 ± 1.05 a	40.43 ± 0.03 b	9053.48 ± 33.97 b	2198.74 ± 22.23 b	47.89 ± 2.25 b	4.06 ± 0.44 a	
	B		14.69 ± 0.46 b	904.96 ± 0.59 a	13,332.62 ± 32.52 a	2682.08 ± 9.07 a	246.34 ± 1.92 a	3.69 ± 0.15 a	
*V.vinifera*	A	37.03 ± 2.47 b	8.40 ± 0.34 a	196.01 ± 2.01 b	882.27 ± 0.66 b	1418.44 ± 1.68 a	7.90 ± 1.01 b	2.11 ± 0.23 a	26.57 ± 0.36 a
	B	45.68 ± 2.06 a	7.50 ± 1.09 a	208.73 ± 1.87 a	1898.58 ± 1.18 a	405.27 ± 0.38 b	9.27 ± 1.19 a	2.15 ± 0.07 a	26.26 ± 0.02 a
*Z.mays*	A	25.03 ± 0.48 b	19.77 ± 0.58 b	444.02 ± 1.66 a	2431.91 ± 9.13 b	2600.47 ± 6.38 a	14.72 ± 1.27 a	3.91 ± 0.22 a	54.99 ± 0.26 b
	B	35.28 ± 0.67 a	24.01 ± 1.85 a	316.35 ± 1.55 b	4076.6 ± 2.97 a	2198.58 ± 6.52 b	11.26 ± 1.79 b	3.63 ± 0.22 b	67.77 ± 0.99 a

Note: Different lowercase letters indicate significant differences in the same physiological and biochemical index of seeds (*p* < 0.05). REC: relative conductivity; MDA: malondialdehyde; CAT: catalase; POD: peroxidase; SOD: superoxide dismutase; α-AA: α-amylase; PRO: soluble protein; TTCH: Dehydrogenase; A: control group; B: direct freezing; C: stepwise freezing; D: vitrification.
